# *Helicobacter pylori* senses bleach (HOCl) as a chemoattractant using a cytosolic chemoreceptor

**DOI:** 10.1371/journal.pbio.3000395

**Published:** 2019-08-29

**Authors:** Arden Perkins, Dan A. Tudorica, Manuel R. Amieva, S. James Remington, Karen Guillemin

**Affiliations:** 1 Institute of Molecular Biology, University of Oregon, Eugene, Oregon, United States of America; 2 Departments of Pediatrics and of Microbiology & Immunology, Stanford University School of Medicine, Stanford, California, United States of America; 3 Department of Physics, University of Oregon, Eugene, Oregon, United States of America; 4 Humans and the Microbiome Program, CIFAR, Toronto, Ontario, Canada; Max-Planck-Institut fur terrestrische Mikrobiologie, GERMANY

## Abstract

The gastric pathogen *Helicobacter pylori* requires a noncanonical cytosolic chemoreceptor transducer-like protein D (TlpD) for efficient colonization of the mammalian stomach. Here, we reconstituted a complete chemotransduction signaling complex in vitro with TlpD and the chemotaxis (Che) proteins CheW and CheA, enabling quantitative assays for potential chemotaxis ligands. We found that TlpD is selectively sensitive at micromolar concentrations to bleach (hypochlorous acid, HOCl), a potent antimicrobial produced by neutrophil myeloperoxidase during inflammation. HOCl acts as a chemoattractant by reversibly oxidizing a conserved cysteine within a 3His/1Cys Zn-binding motif in TlpD that inactivates the chemotransduction signaling complex. We found that *H*. *pylori* is resistant to killing by millimolar concentrations of HOCl and responds to HOCl in the micromolar range by increasing its smooth-swimming behavior, leading to chemoattraction to HOCl sources. We show related protein domains from *Salmonella enterica* and *Escherichia coli* possess similar reactivity toward HOCl. We propose that this family of proteins enables host-associated bacteria to sense sites of tissue inflammation, a strategy that *H*. *pylori* uses to aid in colonizing and persisting in inflamed gastric tissue.

## Abstract 2

A pathogenic bacteria is found to be attracted to bleach. Helicobacter pylori, notorious for causing stomach ulcers and cancer, senses bleach as an attractant, enabling it to localize to sites of inflamed gastric tissue (hypochlorous acid, the active agent of bleach, is produced by host neutrophils as an antimicrobial).

## Introduction

*Helicobacter pylori* is a bacterial pathogen and persistent colonizer of the human stomach and can cause gastritis, ulcers, and stomach cancer [1]. The health burden caused by *H*. *pylori* is particularly large because it infects about half the world’s population, with nearly 100% infection rates in some developing regions, and drug resistance to first-line antibiotics is increasing [1,2]. Despite triggering a robust inflammation response and bursts of reactive oxygen species (ROS) from immune cells, *H*. *pylori* avoids clearance and persists for many decades [3]. The chronic inflammation induced by *H*. *pylori* infection is thought to be a major factor in causing disease [4]. Not only is *H*. *pylori* not eradicated by inflammation, the pathogen, in fact, has been shown to navigate to sites of injury and may capitalize on tissue damage by using host iron extracted from blood hemoglobin and transferrin [5–8].

**Fig 1:**
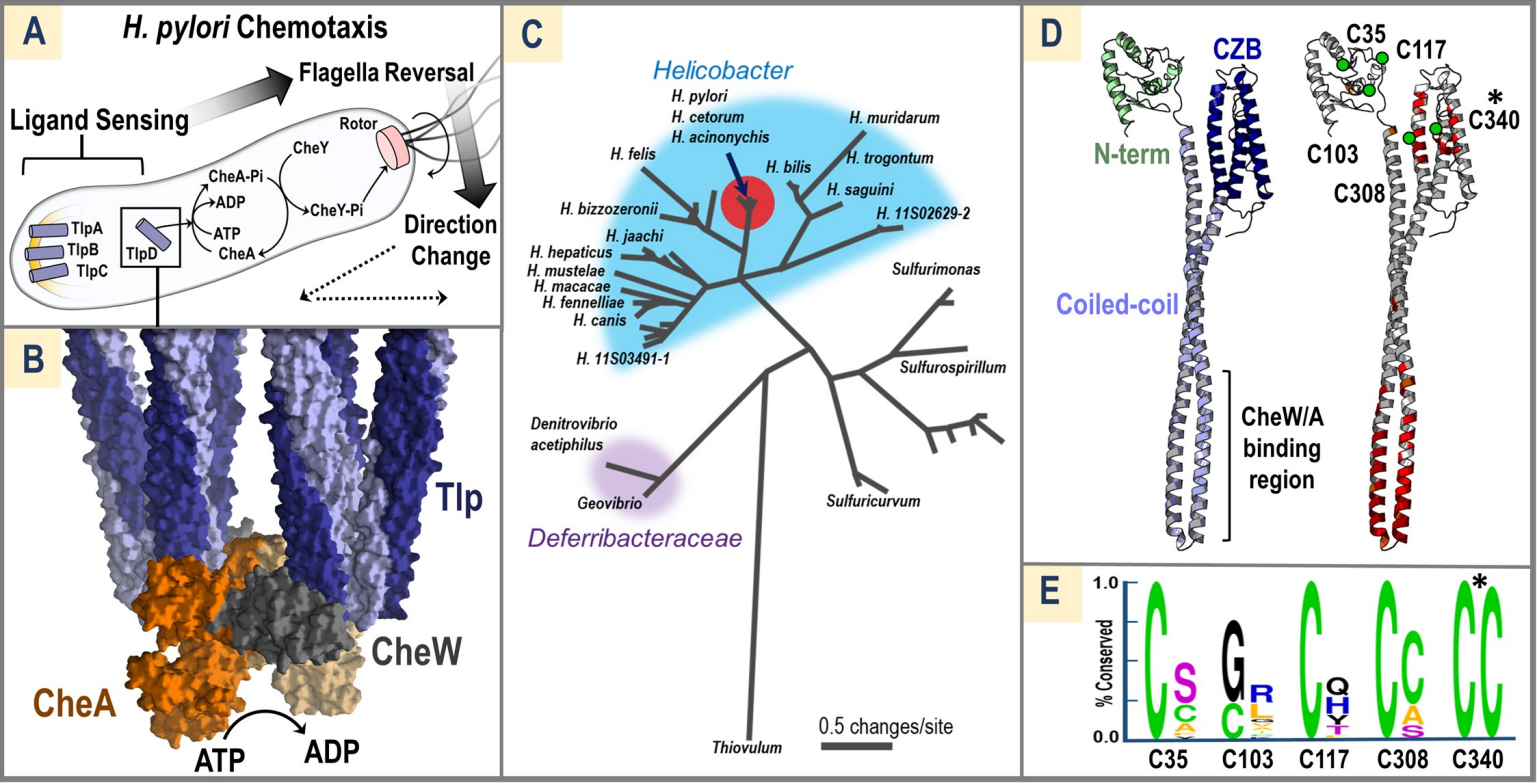
Conservation and Architecture of TlpD. (A) *H*. *pylori* inner-membrane–bound (yellow arc) chemoreceptors TlpA-C and cytosolic TlpD control the autophosphorylation of CheA to CheA-Pi, which then transfers the phosphate to CheY, and CheY-Pi can interact directly with the flagella rotor (pink) to cause a temporary reversal in flagellar rotation. Flagella reversals cause direction changes in swimming trajectory (dotted lines). Black arrows show signal transduction for a chemorepellent response. (B) Two sets of chemoreceptor trimers of homodimers (light and dark blue, denoted as Tlp) associate with the scaffold protein CheW (gray) and a CheA dimer (light and dark orange) to form the core signaling unit and modulate CheA autophosphorylation (illustrative model based on PDB code: 3ja6)[16]. (C) A relatedness tree of TlpD protein sequences from *Helicobacter* (blue), Deferribacteraceae (purple), and other species. *H*. *pylori* TlpD (arrow) is nearly identical to species found in dolphins and cheetas (red dot). (D) A theoretical model of a *Hp*TlpD monomer constructed by i-Tasser with an N-terminal region of unknown structure (light green), the canonical coiled-coil domain of chemoreceptors that interfaces with CheW and CheA (light blue), and a CZB domain (dark blue). Sequence conservation among 459 TlpD homologues is mapped onto the model with 100% conservation highlighted in red and >95% conservation in orange. Cys residues present in the SS1 strain of *Hp*TlpD are noted with green circles. See Zähringer and colleagues [18] for a theoretical model of the TlpD dimer. (E) Conservation of each Cys residue in TlpD is shown with 2 Weblogo plots [19]; the left shows conservation among all sequences of *Hp*TlpD (394 sequences, >92% sequence coverage, >98% sequence identity), and the right plot is conservation among nonpylori TlpD sequences (68 sequences, >91% coverage, >40% sequence identity). Only C340 is universally conserved among all TlpD homologues (noted with * in panels E and D). CheA, chemotaxis protein A; CheA-Pi, phosphorylated CheA; CheW, chemotaxis protein W; CheY, chemotaxis protein Y; CheY-Pi, phosphorylated CheY; CZB, chemoreceptor zinc-binding; *Hp*TlpD, *Helicobacter pylori* TlpD; PDB, Protein Data Bank; TlpA-C, transducer-like proteins A-C; TlpD, transducer-like protein D.

*H*. *pylori* utilizes chemotaxis to seek sites optimal for growth and colonization within the hostile environment of the stomach [9–11](Fig 1A). Bacterial chemotaxis involves a well-studied phospho-relay system prevalent across bacteria and archaea, and functions through a conserved mechanism [12]. Chemoreceptor proteins typically possess a periplasmic ligand-sensing domain to recognize small molecules and transduce signals across the inner membrane to a cytosolic coiled-coil region to regulate ATP-dependent autophosphorylation of the histidine kinase CheA [13] (Fig 1A). Typically, as the cellular pool of phosphorylated CheA (CheA-Pi) is increased, the flagella rotors frequently reverse and alter the swimming bacterium’s trajectory, leading to chemorepulsion; decreases in CheA-Pi result in smooth swimming and chemoattraction (Fig 1A). Chemoreceptors in *H*. *pylori* and other bacteria oligomerize to form trimers-of-receptor dimers (Fig 1B) to build repeating hexagonal arrays that serve to amplify ligand-induced signals up to 50-fold, whereby a single activated receptor can initiate the signal transduction cascade [13–15]. The minimal core unit for signaling is thought to contain two trimers-of-receptor dimers, a CheA dimer, and two CheW scaffold proteins [14,16,17] (Fig 1B).

*H*. *pylori* chemotaxis is mediated by four chemoreceptors, transducer-like proteins (Tlps) A-D (Fig 1A). Environmental chemicals that elicit a chemoresponse by *H*. *pylori* include acid as a chemorepellent [9,20] and urea as a chemoattractant [21], which both serve to direct *H*. *pylori* from the stomach lumen to the gastric epithelium. Tlp deletion mutants have implicated certain chemoreceptors in cellular sensing processes, but only two chemoeffectors have been confirmed in biochemical experiments to act through direct binding to a receptor: TlpB sensing of urea [20,21] and TlpC sensing of lactate [22], though the latter receptor is not conserved and apparently dispensable. Some progress has been made for understanding direct ligand sensing by TlpA, for which recent crystal structures suggest binding of a small hydrophobic ligand [20]. Sensing of pH remains more complicated to understand, because genetic knockouts indicate signals are integrated from TlpA, B, and D [9,20], and no direct sensing mechanism is known for TlpD.

Interestingly, previous work has shown that the cytosolic chemoreceptor TlpD is most highly expressed in *H*. *pylori*, constituting about half of the total chemoreceptor pool [20] (Fig 1A). Such cytosolic or “soluble” chemoreceptors are widespread and common, accounting for approximately 15% of all bacterial and 45% of archaeal chemoreceptors, but their functions are mostly unknown, and to date only a few cytosolic chemoreceptors have been mapped to their ligands [23]. Like membrane-bound homologues, cytosolic chemoreceptors form nanoarrays [24] and therefore represent an intriguing, fully soluble model system to better understand chemoreceptor function. No study has yet described the assembly of cytosolic signaling units in terms of the dissociation constants or reaction kinetics for a cytosolic receptor with CheW and CheA.

A clear consensus on the molecular function of TlpD has remained elusive. Single receptor knockout strains of *H*. *pylori* show defects in colonization of the stomach antrum, a preferred niche for the bacterium and the region of highest inflammation, and of these, *tlpD* strains are by far the most impaired [10]. The first molecular study of TlpD revealed the protein to possess a novel chemoreceptor zinc-binding (CZB) domain that is prevalent among chemoreceptors and diguanylate cyclases of numerous bacteria [25]. The domain uses a rare 3His/1Cys motif to coordinate the bound zinc with high affinity, and the zinc cannot be removed even by the strong zinc chelator N,N,N′,N′-tetrakis(2-pyridinylmethyl)-1,2-ethanediamine (TPEN) [25]. Crystal structures of a wild-type and Cys→Ala mutant CZB domain were solved for diguanylate cyclase Z (DgcZ) (previously called YdeH) from *Escherichia coli*. Those authors suggested CZBs function as zinc sensors and showed for DgcZ that the zinc could be chelated out only by millimolar concentrations of ethylenediaminetetraacetic acid (EDTA) [18]. Additionally, studies using chemotaxis assays to monitor *H*. *pylori* swimming patterns have implicated TlpD as a redox sensor, suggesting it responds to extracellular sources of ROS. However, there are discrepancies in the literature regarding this point. Using the rate of swimming reversals as a metric for chemotaxis, recent work reported a TlpD-dependent increase in reversals from paraquat-generated superoxide [26]. A different study observed a decrease in reversals with superoxide [27]. Additionally, millimolar concentrations of hydrogen peroxide (H_2_O_2_) were reported to increase reversal rates [26], whereas in vivo concentrations of H_2_O_2_ are thought to rarely exceed the low micromolar range [28,29]. TlpD sensing of ROS was also suggested to function as an “energy sensor” by responding to cytosolic oxidants produced through metabolism [27,30]. However, a molecular mechanism has yet to be proposed for how TlpD may function as a sensor.

Here, we have taken advantage of the solubility of TlpD to reconstitute the full (TlpD, CheW, CheA) chemotaxis signaling complex in vitro to directly assay equilibrium and kinetic parameters and to test the effects of putative ligands on receptor signaling. We show TlpD is active and promotes CheA autophosphorylation without the addition of any ligand. Our biochemical characterization of TlpD provides no evidence to support direct sensing of zinc, pH, hydrogen peroxide, or superoxide. Instead, we show that in vitro the strong oxidant hypochlorous acid (HOCl, bleach), the major oxidative product generated by neutrophilic inflammation, potently and reversibly inactivates the signaling complex through a universally conserved cysteine in the TlpD CZB to elicit a chemoattractant signaling response, and that reactivity toward HOCl is a conserved feature of CZB domains from other bacteria. We demonstrate a mechanism by which the cysteine forms a redox “Cys-Zn switch” that is tuned to be reactive toward HOCl and much less reactive toward H_2_O_2_ and superoxide. Lastly, we perform in vivo assays that show *H*. *pylori* tolerates millimolar concentrations of HOCl and uses TlpD for chemoattraction responses to HOCl at physiologically relevant concentrations. We propose this mechanism has evolved to facilitate *H*. *pylori* chemoattraction to sites of inflammation and persistence in neutrophil-rich gastric glands.

## Results

### TlpD homologues possess a universally conserved Zn-binding cysteine

To identify conserved regions important to TlpD function, we performed a protein sequence BLAST search to retrieve putative homologues [31]. This search revealed that TlpD is almost exclusively found in the family Helicobacteraceae, with most homologues being from mammal-associated strains and a few TlpDs from reptile-associated strains (Fig 1C). As expected, TlpD sequences from *H*. *pylori* were highly similar, with >98% sequence identity across 394 isoforms in the nonredundant sequence database. To better understand sequence conservation on a structural level, the i-Tasser program [32] was used to generate a homology model of the receptor monomer, because no experimental structure for TlpD is available (Fig 1D). This model predicts three general domains: an N-terminal region of low-sequence conservation and unknown function, the canonical chemoreceptor coiled-coil that interacts with CheW and CheA, and a C-terminal CZB domain. Mapping positions of high conservation onto the model reveals that the CheW/CheA interface (common to all chemoreceptors) is highly conserved, as are positions in the CZB domain near the Zn-binding residues (Fig 1D). *H*. *pylori* TlpD is unusual for a chemoreceptor, because it contains an abundance of Cys residues (4–5 depending on the strain): C35, C103, C117, C308, and C340 (Fig 1D). Four of the Cys positions are conserved among *H*. *pylori* TlpDs, but only C340 within the CZB domain is conserved across all homologues (Fig 1E).

### Reconstituted chemoreceptor signaling complex retains function in vitro

**Fig 2:**
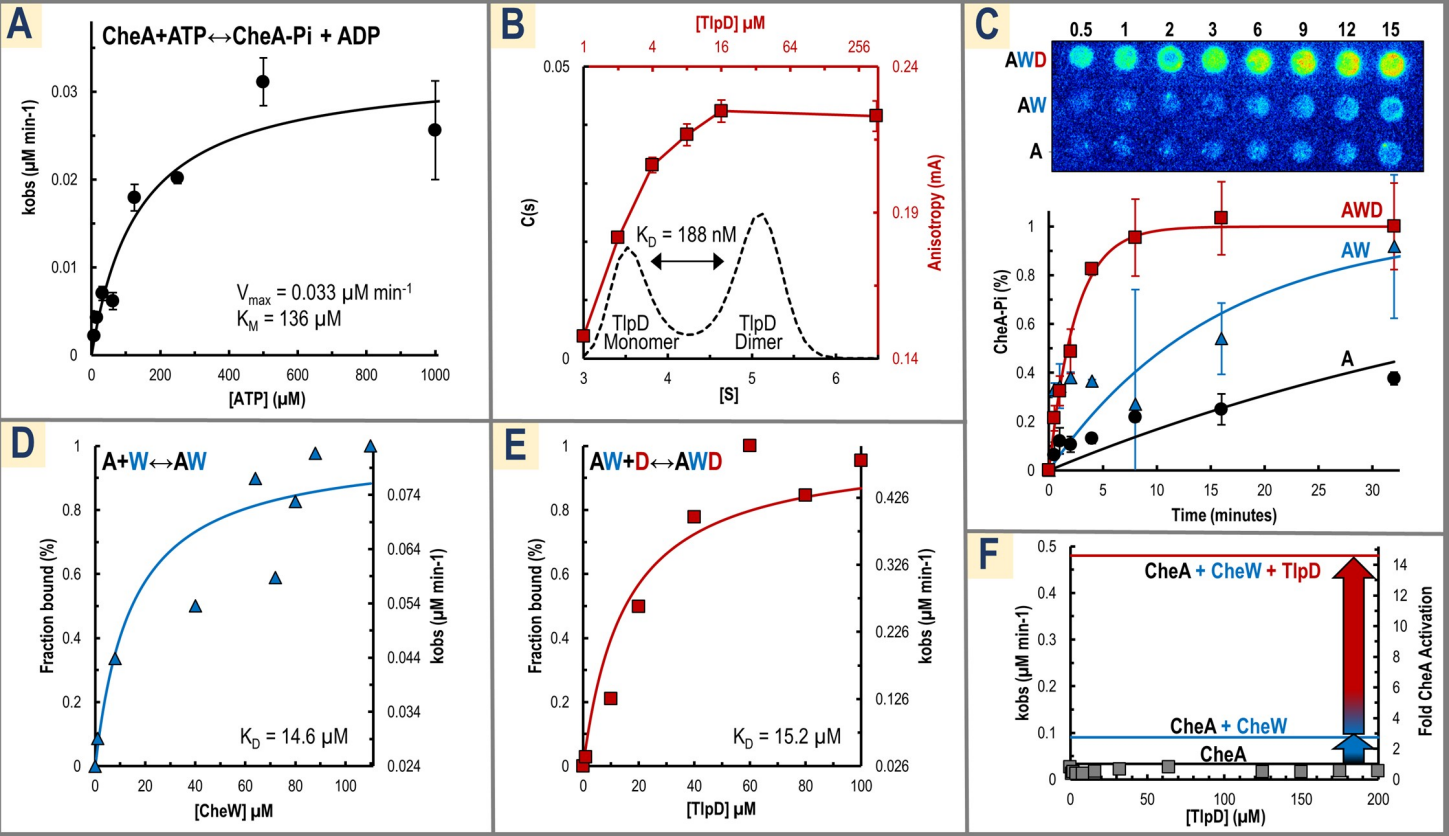
In vitro reconstitution of the TlpD, CheW, and CheA chemotaxis signaling complex. (A) Kinetics of *Hp*CheA with varying concentrations of ATP are shown. Experiments were conducted in triplicate with 4 μM CheA and varying amounts of ATP in 50 mM Tris (pH 7.5), 100 mM NaCl, and 10 mM MgCl_2_. The average k_obs_ for three replicate time courses are shown at various concentrations of ATP (black dots), error bars are the sample standard deviation, and these measurements are fit to the Michaelis-Menten curve (black line). (B) Representative analytical ultracentrifugation data (black axes) are shown for TlpD at 1 μM (black dotted black line) in PBS buffer (pH 7) with 1 mM TCEP. At this concentration, peaks corresponding to the TlpD monomer and dimer occur near 3.5 [S] and 5.2 [S], respectively. The average dimer K_D_ for the recombinant TlpD construct calculated across various protein concentrations was found to be 188 nM (S1 Table). Shown in red on secondary axes are fluorescence anisotropy data for a titration of TlpD under identical conditions with experiments run in triplicate. See also S1 Fig. for a comparison of the TlpD dimer K_D_ with the N-terminal His tag present or cleaved off and simulated data showing the expected monomer and dimer populations expected based on measured K_D_ values. (C) Shown on top are representative raw data from radio-ATP labeling experiments of 15-minute reactions with CheA alone (“A”) and additions of CheW (“AW”) and TlpD (“AWD”). Below are reactions of 1 mM ATP and 4 μM CheA (black circles), +8 μM CheW (blue triangles), and +8 μM CheW; 24 μM TlpD (red squares) run in triplicate and fit to a pseudo–first-order reaction curve (solid lines). (D) CheW was titrated against 4 μM CheA and resulting k_obs_ measurements were fit to a binding isotherm to estimate a kinetically-defined K_D_ of 14.6 μM for the CheA↔CheW interaction. (E) TlpD was titrated against 4 μM CheA and 40 μM CheW and fit to a binding isotherm as in panel C to approximate the thermodynamics of the CheA, CheW↔TlpD interaction to have a K_D_ of 15.2 μM. (F) A titration of TlpD against 4 μM CheA shows no activation (gray squares). For CheA in the presence of saturating [CheW], a 2.7-fold activation occurs (blue line), and with saturating [CheW] and [TlpD], this is increased to a 14.6-fold activation (red line) over CheA alone (black line). See S1 Table for a summary of reaction parameters and statistics. CheA, chemotaxis protein A; CheA-Pi, phosphorylated CheA; CheW, chemotaxis protein W; *Hp*CheA, *Helicobacter pylori* CheA; S, Sedverg; TCEP, tris(2-carboxyethyl)phosphine; TlpD, transducer-like protein D.

Recombinant *H*. *pylori* TlpD, the scaffold protein CheW, and the histidine kinase CheA were expressed and purified for use in functional assays to directly test the effects of mutations and potential ligands using radio-ATP labeling to monitor autophosphorylation of CheA (see Method details). In this assay, activation of CheA increases CheA autophosphorylation which promotes swimming reversals and chemorepulsion; decreasing CheA activity increases smooth swimming and chemoattraction (Fig 1A). Autophosphorylation kinetics of *Hp*CheA alone revealed a K_M_ for ATP of 136 μM, similar to the approximately 300 μM K_M_ reported for *E*. *coli* CheA; however, the basal level of k_cat_ was exceptionally slow at 8.25 × 10^−3^ min^−1^ (compared with 1.56 min^−1^ for *E*. *coli*)[33](Fig 2A). We next determined the concentration of TlpD required to achieve maximal dimerization, because the receptor dimer is the core building block for the chemotaxis signaling complex [17]. The dimer K_D_ of our TlpD recombinant construct was found by analytical ultracentrifugation to be 188 nM, consistent with fluorescence anisotropy experiments that showed that at approximately 16 μM and above TlpD is mostly dimerized (Fig 2B and S1 Fig). Based on these results, assays with TlpD were generally run at concentrations of 20 μM or higher so that the properties of the physiologically relevant dimer were observed rather than the inactive monomer. Addition of stoichiometric concentrations of TlpD, CheW, and CheA resulted in synergistic activation of the chemotaxis complex; CheW was able to partially activate CheA alone, and activation was further increased with the addition of TlpD (Fig 2C). These results confirmed that the recombinant system was functional and activated CheA in a manner similar to other described chemotaxis systems [34].

We next leveraged this system to estimate kinetically defined dissociation constants for the components and to determine the required order of complex assembly using radio-ATP labeling. Titration of CheW against CheA revealed an estimated K_D_ of 14.6 μM for formation of the CheW-CheA complex, similar to that of *E*. *coli* at 17 μM [35], and maximal activation of 2.7-fold (Fig 2D). Titration of TlpD against CheW-CheA showed an estimated K_D_ of 15.2 μM and maximal 14.6-fold activation of the complex (Fig 2E). Previous work has shown for *E*. *coli* that CheW is required for the receptor to activate CheA [36], but a recent study suggested TlpD could activate CheA independently [37]. However, that study used nonstoichiometric ratios for the chemotaxis components and only 2 μM TlpD; based on our measurements of the TlpD dimer K_D_, approximately 12% to 20% of the receptor could be in its inactive monomer form at this concentration (S1 Fig). Direct activation of CheA by TlpD was tested with a titration of TlpD against CheA without CheW, but in our hands no activation occurred even at 200 μM receptor (Fig 2F). Instead, the data support a sequential activation of the complex whereby CheA and CheW first associate to achieve 18% activation, and then TlpD can bind and promote full 100% activation of the chemotaxis complex (Fig 2F). To our knowledge, this is the first characterization of dissociation constants and autophosphorylation rates of a cytosolic chemotaxis complex and indicates in this case that the chemoreceptor TlpD is “on” by default and able to activate CheA even without the addition of other ligands. Kinetic parameters and dissociation constants are summarized in S1 Table.

### TlpD is not directly sensitive to exogenous Zn^++^, pH, hydrogen peroxide, or superoxide

Two lines of evidence led us to hypothesize that the zinc atom bound to the CZB domain might be a cofactor in ligand sensing. First, due to its resistance to zinc chelation, TlpD was previously estimated to bind zinc with subfemtomolar affinity [38], which seems inconsistent with a sensor that is activated by exogenous zinc, considering that ligand affinity in other bacterial chemoreceptors is typically in the micromolar range [12,20,39]. Inductively coupled plasma (ICP) mass spectrometry analysis of recombinantly-grown TlpD previously showed TlpD to be zinc-loaded following purification without addition of zinc [25]. We verified that addition of 6 to 24 μM zinc (0.25× to 1× concentration relative to TlpD) to the functional assay did not substantially impact autophosphorylation rates, and higher concentrations, far beyond those considered to be physiologically relevant, caused the proteins to precipitate (S2A Fig). To further test the feasibility of a reversible zinc-sensing function, we attempted to remove zinc from TlpD by chelation with the fluorescent probe Zinpyr-1, which binds zinc with nanomolar affinity, but no chelation was observed, in support of earlier work by Draper and colleagues [38] (S2B Fig and S2C Fig). We also heat-denatured TlpD, and only a submolar equivalent of zinc was released, even after 3 hours of incubation with Zinpyr-1 (S2C Fig). Circular dichroism (CD) of the protein solution indicates that the lack of chelation is not due to the protein refolding (S2D Fig), suggesting that even when the polypeptide is denatured, zinc is sufficiently buried to prevent removal. Given the absence of data demonstrating the reversibility of zinc binding or impact on complex signaling activity, TlpD may not directly detect exogenous Zn^++^ as a chemotaxis ligand.

**Fig 3:**
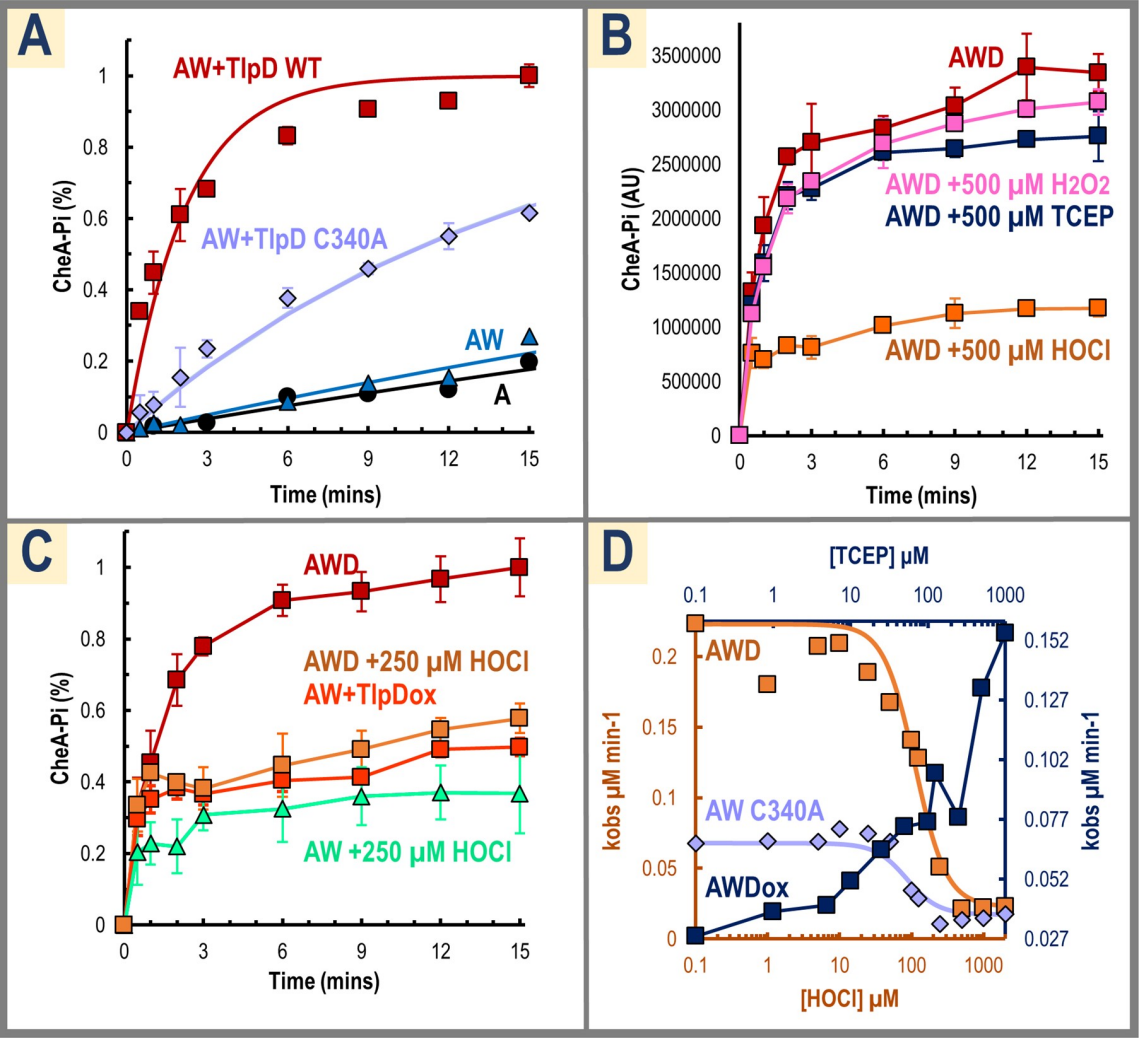
Chemotaxis signaling responses to ROS. (A) Reaction time courses with reconstituted signaling complex are shown using either wild-type TlpD (CheW and CheA present, red squares) or C340A (CheW and CheA present, violet diamonds), with single time courses for CheA alone (black circles) and CheA and CheW (blue triangles) for reference. Data were run in triplicate and are fit with pseudo–first-order curves (solid lines). (B) Shown are functional assay time courses following 1-H pretreatments at pH 7 with either PBS buffer (red), 500 μM H_2_O_2_ (pink), TCEP (dark blue), or hypochlorous acid (HOCl, orange), using TlpD, CheW, and CheA (*n* = 3). Chemoeffectors were diluted in PBS buffer (pH 7). (C) Functional assays are shown with 250 μM HOCl either added directly to the reconstituted complex (CheW and CheA present, light orange boxes), using preoxidized TlpD (CheW and CheA present, dark orange boxes), or HOCl treatment of CheA and CheW alone with no TlpD added (light green triangles), relative to the untreated reconstituted signaling complex (TlpD, CheW, and CheA, red boxes; *n* = 3). Samples were treated with HOCl or buffer as indicated for 30 minutes and subsequently quenched with 1 mM methionine prior to addition of other reaction components. (D) A series of functional assays with reconstituted signaling complex titrating with HOCl is shown with k_obs_ values from reaction time courses plotted against [HOCl] on a log scale (TlpD, CheW, and CheA, orange boxes). Fitting these data to a binding isotherm with a Hill coefficient of 2 yields a K_1/2_ for inhibition of TlpD-activation of CheA activity by HOCl of 110 μM (orange line). An identical titration was performed using the C340A mutant (violet diamonds and line). Recovery of inactivated complex with wild-type TlpD by reduction with TCEP is shown in dark blue and plotted on a secondary axis. Complex was pretreated for 1-H with 125 μM HOCl and then treated with varying [TCEP] for 30 minutes. See also S2 Fig. for additional analyses of responsiveness to zinc, pH, and paraquat. AW, complex formed by CheA and CheW; AWD, complex formed by CheA, CheW, and TlpD; CheA, chemotaxis protein A; CheA-Pi, phosphorylated protein CheA; CheW, chemotaxis protein W; K_1/2_, the concentration at which 50% of the kinase was inactivated; ROS, reactive oxygen species; TCEP, tris(2-carboxyethyl)phosphine; TlpD, transducer-like protein D.

The crystal structure of the *E*. *coli* CZB, in which the cysteine residue equivalent to the conserved TlpD C340 was mutated to an alanine (Protein Data Bank [PDB] code: 4h54), provides a second line of evidence in favor of Zn as a cofactor in ligand sensing. In this structure, the zinc remained bound by the 3-His core in the absence of the Cys S_γ_ [18], hinting that in the wild-type protein the Cys side chain might be released from the zinc in response to some molecular cue. The importance of C340 in promoting CheA autophosphorylation was demonstrated using our functional assay; compared with wild-type TlpD, the C340A mutant caused a 6.8-fold loss in CheA activation, suggesting that the Cys S_γ_ is required for full activation by TlpD (Fig 3A).

We also explored a potential role of the zinc-binding core of TlpD in direct pH sensing, because protonation of C340 could cause it to dissociate from the bound Zn. In vivo data show TlpD participates in both an acid chemorepulsion and chemoattraction to basic pH [9], and if this mechanism involved direct sensing by TlpD, we would expect this to be reflected in our in vitro functional assay as decreasing CheA activation as a function of increasing pH. However, functional assays across different pH did not replicate in vivo responses (see Method details and S2E Fig). Activity was similar across pH 6.6 to 7.8, the normal range that TlpD would be expected to experience within the well-buffered cytosol [40]. Together, these data led us to conclude that, although TlpD is important for acid sensing in vivo, the receptor apparently does not directly sense pH.

Finally, we tested if C340 might directly sense the oxidants tested previously by Collins and colleagues and Behrens and colleagues [26,27]. Functional assays were performed with 500 μM H_2_O_2_ or 500 μM of the reductant TCEP but no change in activity was observed (Fig 3B). Addition of paraquat at concentrations used in previous *H*. *pylori* chemotaxis assays [27] also did not alter activation of the complex, suggesting TlpD does not directly sense superoxide (S2F Fig).

### TlpD directly senses hypochlorous acid through oxidation of a conserved cysteine

Though previous work had tested TlpD-dependent sensing of the ROS hydrogen peroxide and superoxide [26,27], and *H*. *pylori* is well known to inhabit and persist within inflamed tissue [1], no study had yet analyzed whether TlpD might respond to the inflammation oxidant HOCl. Addition of 500 μM HOCl to reconstituted TlpD-CheW-CheA complex dramatically decreases CheA autophosphorylation to the rates observed for CheW-CheA alone (Fig 3B). Functional assays using preoxidized TlpD show a similar decrease in CheA autophosphorylation as HOCl-treated complex, whereas HOCl treatment of CheW-CheA alone does not impair activity relative to untreated CheW-CheA, suggesting the source of inhibition of signaling complex activity is due to oxidation of TlpD (Fig 3C). A titration with HOCl revealed the concentration at which 50% of the kinase was inactivated, which we refer to as K_1/2_ [41], to be approximately 110 μM (Fig 3D). A similar titration using the C340A mutant exhibited largely diminished activity even without the addition of HOCl, indicating the mutation may effectively mimic the “off” state of the receptor (Fig 3D). In these assays, no protein precipitation was observed, but because HOCl is known to oxidatively damage proteins and cause aggregation [42], HOCl-oxidized samples were tested for resurrection by the reductant TCEP, which was shown to effectively reactivate the complex (Fig 3D).

**Fig 4:**
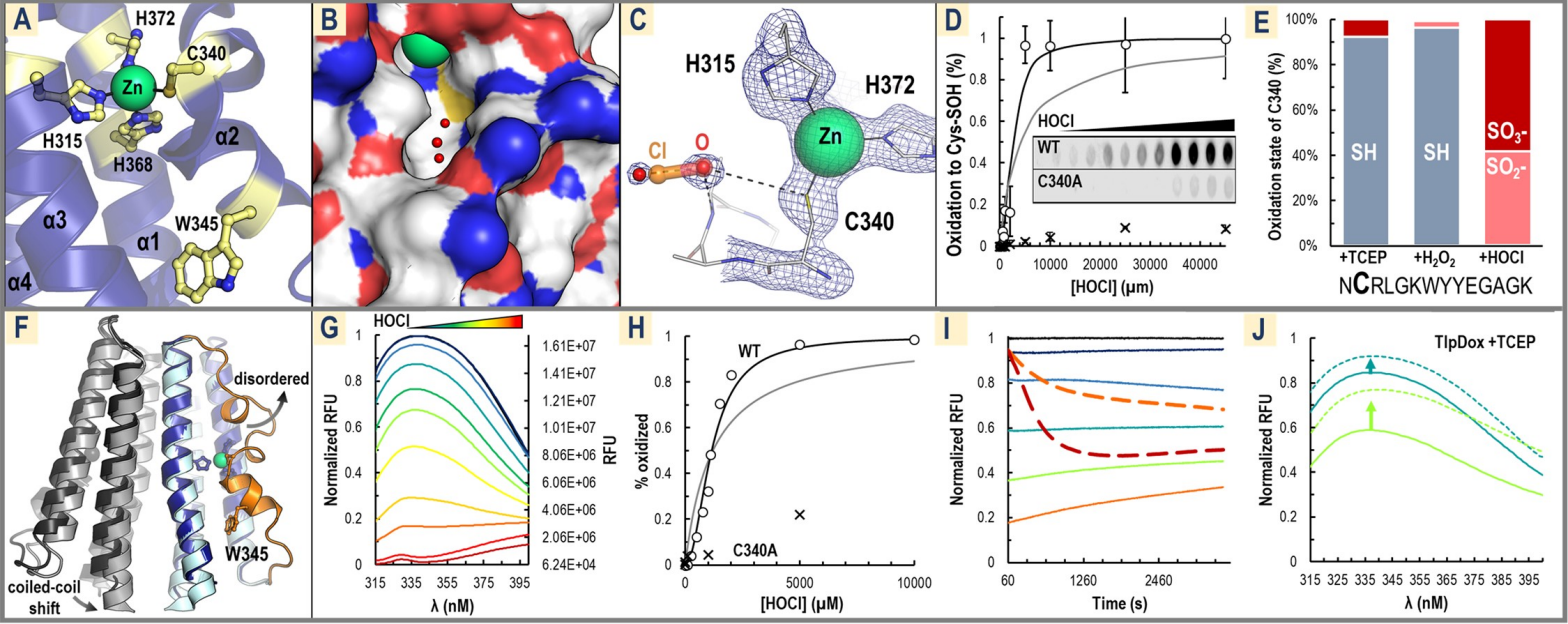
Structural and biochemical analyses of TlpD C340 oxidation. (A) The crystal structure of the *E*. *coli* CZB domain is shown, with the conserved residues of the Zn binding core and nearby W345 highlighted in gold (PDB code: 3t9o, numbering is for corresponding residues in *Hp*TlpD based on sequence alignment). (B) The molecular surface in the same view as in panel A (sulfur is yellow; nitrogen is blue; oxygen is red; carbons are white; zinc is green) showing C340 is accessible to solvent. Three waters in the experimental structure are shown as small red spheres. (C) A close-up view of the CZB crystal structure is shown in which 2 water molecules bind in the pocket near C340, mimicking the predicted position of hypochlorous acid (O and Cl labeled and shown as transparent sticks). 2fo-fc electron density is shown as blue mesh, contoured at 1.5 σ. (D) Oxidation of 20 μM WT TlpD (open circles) or C340A mutant (crosses) to form Cys-SOH is shown at various HOCl treatments and monitored using an anticysteine-sulfenate antibody. Experiments were run in triplicate, and representative raw data are shown in the inset, with the image length reduced by 50%. Fits of the WT data to the Hill equation using either a coefficient of 1 (gray line) or 2 (black line) are shown. S3 Fig. Panel A shows these data plotted on a log scale. See S4 Fig. for similar experiments with the CZB domain of *E*. *coli* DgcZ and *Salmonella* McpA. (E) Data from MS/MS experiments show oxidation states of C340 following treatments of TlpD with 1 mM TCEP, H_2_O_2_, or HOCl. The 14-mer peptide containing C340 (noted in bold) was identified and found to be modified either by cysteine alkylation (gray, SH, indicating no oxidation), oxidation to a cysteine sulfinate (peach, SO_2_^−^), or oxidation to a cysteine sulfonate (dark red, SO_3_^−^). See also S3C–S3E Fig. and Method details. (F) Shown is an overlay of the WT *E*. *coli* CZB homodimer (dark blue, PDB code: 3t9o) and Cys→Ala mutant (light blue, PDB code: 4h54) crystal structures. Without the Cys-Zn bond, the region containing C340 and W345 (orange) locally unfolds and becomes disordered and solvent exposed. This structural rearrangement also promotes an approximately 3 Å shift in its dimer partner (wild-type dark gray, Cys→Ala light gray) in the helix adjoining the coiled-coil that may propagate to the CheW/CheA interface to deactivate the signaling complex. (G) Intrinsic protein fluorescence data (excited at 295 nm) for a titration of HOCl against 20 μM TlpD is shown. Treatments were for 30 minutes with 20 μM TlpD in PBS (pH 7) with addition of 0 μM (black), 100 μM (dark blue), 250 μM (light blue), 500 μM (teal), 750 μM (green), 1 mM (lime green), 1.1 mM (yellow), 1.5 mM (light orange), 2 mM (orange), 5 mM (red), or 10 mM (dark red) HOCl. (H) Data from panel F is quantified with the normalized RFU at 340 nm at each [HOCl] concentration fit to the Hill equation with a coefficient of 1 (gray) or 2 (black). Representative equivalent experiments performed with C340A are shown as crosses. (I) Time courses comparing fluorescence quenching at 340 nm (excited at 295 nm) of 20 μM TlpD by peroxide (2 thick dashed lines) and HOCl (5 thin solid lines) at concentrations of 0 μM, 100 μM, 250 μM, 500 μM, 1 mM, 2 mM, and 5 mM, colored as in panel C. (J) Fluorescence experiments are shown of oxidized TlpD treated with TCEP (excited at 295 nm); 20 μM TlpD samples were preoxidized (solid lines) with HOCl at concentrations of 500 μM (blue) and 1 mM (lime) for 30 minutes prior to reduction by equimolar concentrations of TCEP for 30 minutes (dashed lines), resulting in recovery of fluorescence intensity (upward-pointing arrows). CheA, chemotaxis protein A; CheW, chemotaxis protein W; Cys-SOH, cysteine sulfenic acid; CZB, chemoreceptor zinc-binding; DgcZ, diguanylate cyclase Z; *Hp*TlpD, *Helicobacter pylori* transducer-like protein D; PDB, Protein Data Bank; RFU, relative fluorescence units; TCEP, Tris(2-carboxyethyl)phosphine; TlpD, transducer-like protein D; WT, wild type.

These data suggest the mechanism of HOCl sensing involves oxidation of TlpD C340 to a cysteine sulfenate, an oxidation product commonly formed in redox systems that utilize a redox-active cysteine and that is readily reversible by small molecule and protein reductants such as the thioredoxin/thioredoxin reductase system present in *H*. *pylori* [43,44]. Inspection of the *E*. *coli* CZB structure reveals a small channel that allows solvent to access the cysteine, which has not been previously reported (Fig 4A and 4B). The C340 S_γ_ is partially solvent exposed at the base of this cavity, reminiscent of an enzyme active site suitable for binding a small ligand (Fig 4B). In this site, two water molecules identified in the crystal structure could mimic the approximate positions for an oxygen and chloride of a hypochlorite molecule and are coordinated by the backbone NH of the following helical turn to be well positioned to react with the C340-S_ɣ_ (Fig 4B and Fig 4C). To determine if C340 can react with HOCl and become oxidized to a cysteine sulfenate, we treated wild-type TlpD and C340A mutant protein with HOCl and monitored formation of cysteine sulfenate by dimedone adduction and western blotting [45]. The results show potent formation of cysteine sulfenate for the wild-type protein but almost none for the C340A mutant (Fig 4D, S3A Fig). This indicates not only that HOCl can access and directly oxidize C340 in solution but also that C340 is tuned to be highly reactive with HOCl, as it is approximately 50-fold more readily oxidized than the four other TlpD Cys residues combined (Fig 4D, S3A Fig). Titration data exhibit a sigmoidal response and are fit well with a Hill coefficient of n = 2, suggesting positive cooperativity occurs across the receptor homodimer (Fig 4D and S3A Fig). As a secondary test of direct oxidation of C340 by HOCl, we performed mass spectrometry of reduced, H_2_O_2_-treated, and HOCl-treated TlpD to look for the formation of oxygen adducts. The spectra indicated little oxidation of C340 occurred for the reduced and peroxide-treated samples, but spectra of HOCl-treated TlpD showed C340 to be completely oxidized to sulfinate and sulfonate forms (Fig 4E and S3C–S3E Fig). Similar “overoxidation” in the absence of reductant in in vitro assays is seen to occur for other proteins with strongly redox-active Cys residues [46,47], but bacteria generally lack the capacity to reduce sulfinate and sulfonate forms [48], so we expect that cysteine sulfenate is the oxidation state of physiological relevance. Lastly, we tested two other representative CZBs, the CZB domain of the *E*. *coli* diguanylate cyclase DgcZ and the *Salmonella enterica* chemoreceptor McpA. We found them to be similarly reactive toward HOCl as TlpD, suggesting HOCl sensing is a conserved function of CZB domains (S4 Fig.).

Comparison of the wild type and Cys→Ala CZB crystal structures shows that in the mutant a region containing 22 residues, including the position of the conserved cysteine, locally unfolds and becomes disordered (Fig 4F). We speculate that oxidation of C340 would promote a similar conformational change, leading to signal transduction by shifting the upstream coiled-coil region (Fig 4F). Fortuitously, TlpD contains a single conserved Trp residue (W345) located near C340 that should participate in this conformation change (Fig 4A and Fig 4F). Therefore, fluorescence of W345 was used as a native probe to detect the receptor’s conformation change in response to C340 oxidation. Strong Trp fluorescence is observed in untreated TlpD samples but is effectively quenched by HOCl concentrations in the μM to mM range, supporting that oxidation induces a conformation change like that observed in the crystal structures (Fig 4G). Similar to the apparent cooperativity observed for oxidation of C340 (Fig 4D), the conformation change exhibits a sigmoidal response (Fig 4H). The C340A mutant is about 5-fold less sensitive to HOCl treatment than wild type, showing this conformation change is dependent on the presence of C340 (Fig 4H). To ensure that loss of fluorescence was not due to protein denaturing, a similar titration was monitored by CD, which shows TlpD remains folded up to at least 10 mM HOCl and partially folded even at 100 mM HOCl (S3B Fig). Trp fluorescence quenching time courses revealed HOCl reacts to completion within the sample mixing time (<60 seconds), whereas quenching by hydrogen peroxide requires 6- to 7-fold higher concentrations and is very slow, with the reaction requiring approximately 20 minutes to reach completion (Fig 4I). Quenched fluorescence of oxidized TlpD was recovered by reduction with TCEP (Fig 4J). Together, these data support a model in which HOCl oxidation of TlpD C340 stimulates a conformational change in the receptor, although further analysis will be required to understand how these structural changes may regulate the distant CheA interface.

### Hypochlorous acid decreases *H*. *pylori* swimming reversals

**Fig 5:**
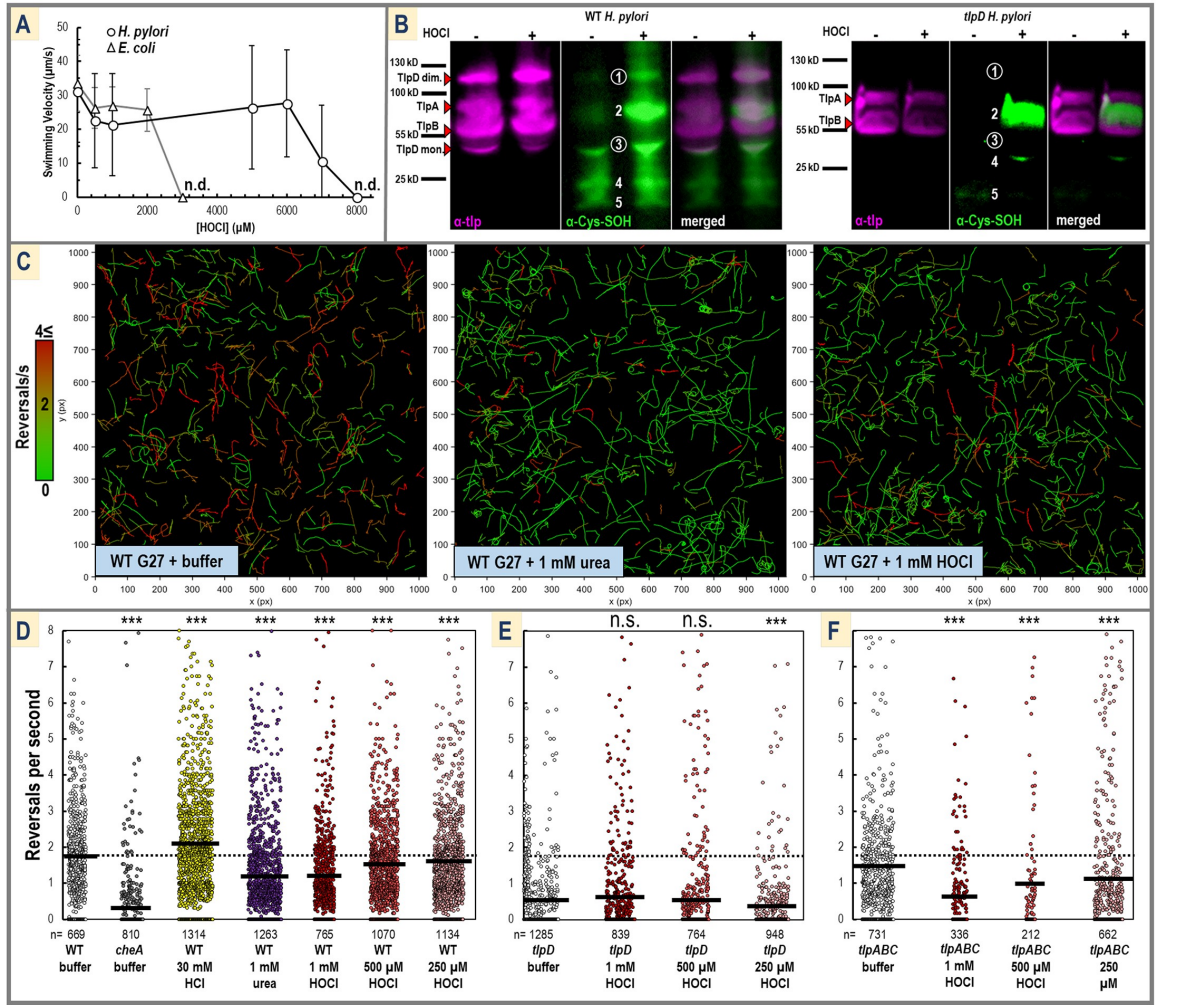
*H*. *pylori* swimming behavior in response to HOCl treatment. (A) Shown is the mean swimming velocity following treatment with various concentrations of HOCl using the chemotaxis-null *E*. *coli* strain UU1250 (triangles, gray line) and *H*. *pylori cheA* (circles, black lines). The concentration of HOCl at which no motile bacteria were detected is noted with “n.d.” Error bars shown are the sample standard deviation. Experiments were performed in triplicate, with *n* > 30 bacteria tracks (see Method details). (B) Shown are representative western blots of WT and *tlpD H*. *pylori* cell lysate from cells treated with either PBS buffer (pH 7) or 1 mM HOCl in PBS buffer (pH 7). Cells from liquid cultures were treated for 30 seconds in the presence of dimedone, then quenched by methionine to inactivate residual HOCl. Blots of cell lysate were probed with α-Tlp primary antibody (pink), stripped and re-probed with α-cysteine-sulfenate primary antibody (green), and visualized with chemiluminescence. Bands appearing in the α-Tlp blot corresponding to the molecular weights of TlpA, TlpB, and TlpD monomer and disulfide-linked dimer are noted with red triangles. Five prominent bands in the α-cysteine-sulfenate blot of WT *H*. *pylori* are numbered, with bands 1 and 3 noted with circles that appear at the same molecular weight as the TlpD monomer and dimer bands. Bands 1 and 3 are absent in the *tlpD* mutant. Images shown are false-colored and overlaid and merged with ImageJ. Data shown in this panel are from lanes 4 and 5 of the full blots; see S5 Fig and S6 Fig for full blots with 4 to 5 independent experiments for each treatment and Method details. (C) Representative swimming tracks of WT *H*. *pylori* are shown following the addition of PBS buffer (pH 7), 1 mM urea in PBS buffer (pH 7), or 1 mM HOCl in PBS buffer (pH 7). Tracks are colored according to reversal rate on a spectrum of green (low) to red (high). In these figures, 1 pixel corresponds to 0.65 microns. Note that these images are intended to visualize the swimming behavior of the bacterial swarm but that individual reversals cannot be seen at this scale. (D–F) Reversal rates for WT and mutant *H*. *pylori* are shown for various treatments. Experiments were conducted in triplicate, and each dot corresponds to a single swimming bacteria track captured during a 30 second video, with the mean reversal rate for each treatment indicated with a dark black horizontal line. The baseline reversal rate for the WT in PBS buffer is shown as a dotted black line. For clarity, only data points in the 0 to 8 reversals/second range are displayed. A small percentage of outliers exist outside of this range and are included in the calculation of the mean shown. Because data are not normally distributed, *p*-values were calculated using the nonparametric Kolmogorov-Smirnov two-sample test [50] comparing each data set to WT in buffer (D), *tlpD* in buffer (E), or *tlpABC* in buffer (F). *p* < 0.001 are indicated with *** and *p* > 0.05 are noted as n.s. All experiments were performed using *H*. *pylori* strain G27 or mutants created in that background. See also S7 Fig for the definition of a swimming reversal applied in these analyses and control HOCl treatments with a *cheA* mutant. See S2 Table for numerical mean and distribution values. n.s., not significant; TlpA, transducer-like protein A; TlpB, transducer-like protein B; TlpD, transducer-like protein D; WT, wild type.

HOCl can reach concentrations as high as 5 mM at sites of inflammation [49], and so prior to investigating *H*. *pylori* sensing of HOCl in vivo, we first determined the range of HOCl concentrations *H*. *pylori* can survive and remain motile. We used a video tracking assay to measure swimming velocities in response to acute treatments of HOCl in the absence of chemotaxis for an *H*. *pylori cheA* mutant (strain G27), which is motile but cannot respond to chemotaxis signals, and a motile *E*. *coli* lacking all chemoreceptors (strain UU1250; Fig 5A). We found that *H*. *pylori* is extremely resilient against HOCl treatment, with full loss of motility observed only for treatments exceeding approximately 7 mM, whereas *E*. *coli* lost motility in treatments exceeding 2 mM HOCl (Fig 5A).

Western blot experiments were performed to investigate if exogenous HOCl can enter *H*. *pylori* cells and oxidize cysteine thiols. Liquid cultures of wild-type *H*. *pylori* and a *tlpD* mutant (strain G27) were treated with either PBS buffer or 1 mM HOCl in buffer in the presence of dimedone, which is cell permeable, quenched with methionine, and then cell lysates were examined for the presence of cysteine sulfenate (Fig 5B and S5 and S6 Figs). Western blots of samples were probed with α-Tlp antibody [26], then stripped and reprobed with α-cysteine-sulfenate. The HOCl-treated wild-type cells exhibit approximately five prominent bands corresponding to protein targets that are oxidized by HOCl to form cysteine sulfenate, suggesting that HOCl is sufficiently cell permeable to oxidize cytosolic thiols and that these targets are rather specific (Fig 5B). Interestingly, two of the five cysteine-sulfenate bands in the wild-type blot appear at approximately the same molecular weight as TlpD monomer and disulfide-linked TlpD dimer [27], and these bands are absent in the *tlpD* mutant blot (Fig 5B and S5 and S6 Figs). These data suggest that TlpD is one of only a few protein targets of HOCl cysteine oxidation; however, detecting the oxidation of a single cysteine residue in vivo is technically challenging, and additional analyses are needed to establish the identities of these cysteine-sulfenate protein bands.

We next showed that *H*. *pylori* swimming reversal rates are altered by HOCl exposure. Chemorepellants such as acid stimulate a build-up in CheA-Pi that results in more frequent flagella reversals and an increased rate of direction changes that, here, we refer to as “reversals”; conversely, chemoattractants reduce cellular CheA-Pi to decrease swimming reversal rate and promote smooth swimming [26]. We used automated particle tracking [51] to track almost all motile bacteria from each experiment and characterize their trajectories according to a standard definition for swimming reversal, thereby eliminating observer bias and increasing statistical power over manual tracking methods (see Method details and S7A Fig). Wild-type strain G27 *H*. *pylori* treated with PBS buffer showed a diverse range of reversal rates, with an average reversal rate of 1.77 s^−1^ (*n* = 669; Fig 5C left, S1 Movie). Addition of 1 mM of the chemoattractant urea [21] shifted the swimming behavior to be mostly smooth swimming, reducing the average reversal rate to 1.21 s^−1^ (*n* = 1,263; Fig 5C center, S2 Movie). Addition of 1 mM HOCl similarly promoted smooth swimming and decreased the reversal rate to also be 1.21 s^−1^, consistent with chemoattraction behavior (*n* = 765; Fig 5C right, S3 Movie). In comparison, by our metrics, a chemotaxis-null *cheA* mutant exhibited a much lower reversal rate of 0.32 s^−1^ and was unresponsive to HOCl (S7B Fig, S4 and S5 Movies).

Experiments conducted for three concentrations of HOCl in the range of 250 μM to 1 mM resulted in a dose-dependent decrease in reversal rate for wild type (Fig 5D). Similar assays were performed with a *tlpD* mutant, which exhibited a very low baseline reversal rate, as observed previously by Collins and colleagues and Schweinitzer and colleagues [26,30], and reversals were not further decreased by HOCl treatment (Fig 5E, S6 and S7 Movies). Despite its low reversal rate, previous work has shown *tlpD* mutants are not chemotaxis-null and retain chemoattraction to urea through TlpB [9]; however, quantification of reversal rates is inherently poor at identifying further decreases when the baseline is already near zero. To further investigate if these decreases in reversal rates from HOCl treatments are TlpD-dependent, assays were performed with a *tlpABC* mutant, which possesses TlpD as its sole chemoreceptor, and this mutant showed dose-dependent decreases in reversal rates similar to wild type (Fig 5F, S8 and S9 Movies). Calculations of average reversal rates and sample distributions are listed in S2 Table.

### *H*. *pylori* is attracted to hypochlorous acid

**Fig 6:**
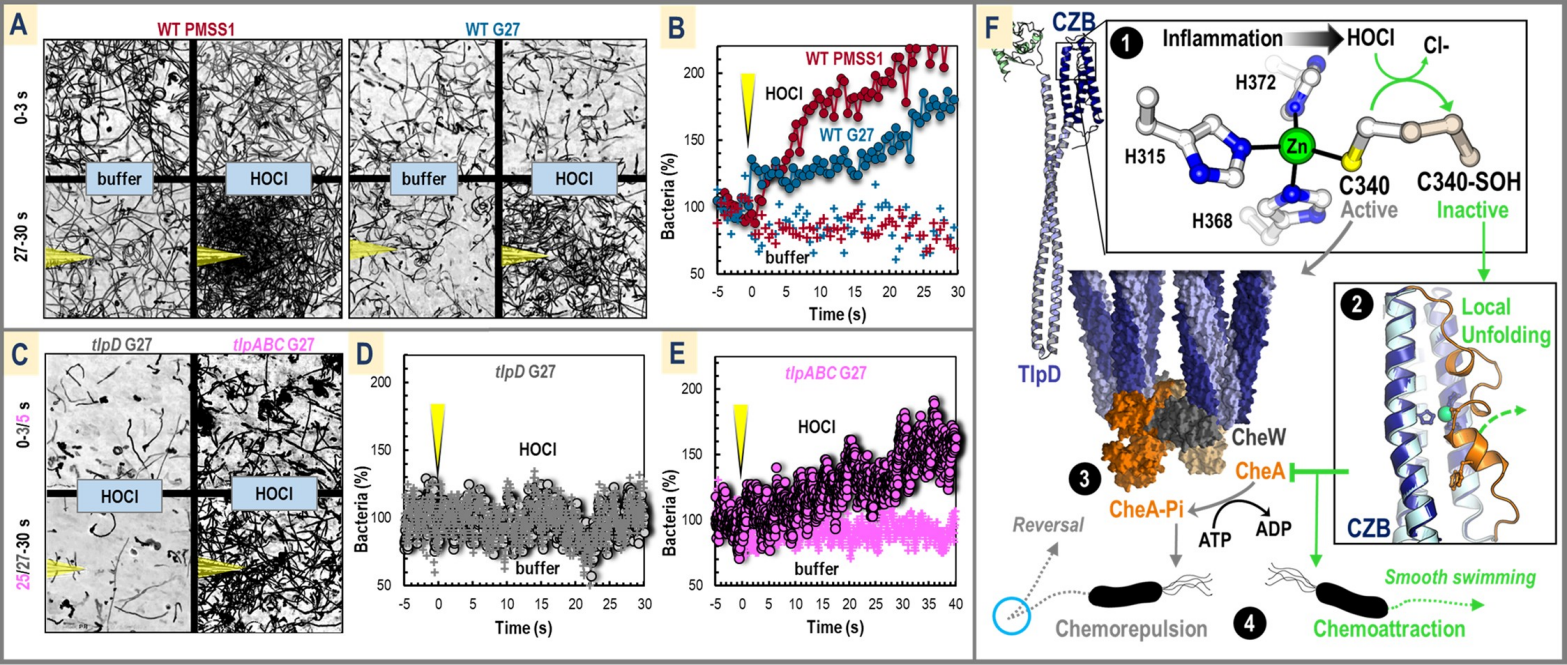
*H*. *pylori* TlpD-dependent chemoattraction to sources of HOCl. (A) Data from point-source chemotaxis experiments [21] are shown for *H*. *pylori* strains PMSS1 (red) and G27 (blue) using a highly calibrated micropipette for chemoeffector delivery. Three-second motility traces are shown prior to treatment (0–3 seconds) and following 30 seconds of treatment (27–30 seconds) with buffer (500 mM sodium phosphate [pH 6.7]) or 10 mM HOCl (diluted into buffer [pH 6.7]). The position of the micropipette is highlighted in yellow in the treatment panels. (B) Data from panel A is quantified over time with the average percentage of bacteria in the field of view prior to treatment normalized to be 100%. Treatment with buffer (pluses) or 10 mM HOCl (circles linked by lines) begins at time zero, noted with the yellow micropipette. Each data point shown is the average over 2 seconds of video (60 frames). (C) Experiments performed as in panel A with HOCl for the *tlpD* G27 (gray, 3-second time traces) and *tlpABC* G27 (pink, 5-second time traces) mutants. (D) Quantified data for *tlpD* ± 10 mM HOCl. (E) Quantified data for *tlpABC* experiments ± 10 mM HOCl. For panels D through E, each data point shown is the normalized bacteria count per frame. See supplementary materials for videos of these experiments and statistical analysis. (F) Shown is a synthesis of the data from this work as the basis for a new model for how TlpD functions to sense HOCl as a chemoattractant. By default, TlpD is active and promotes swimming reversals (blue circle) by stimulating CheA autophosphorylation (gray arrows). TlpD is deactivated by HOCl sensing, leading to smooth swimming and chemoattraction (green arrows). The inflammation product HOCl oxidizes a specially tuned C340 to form a cysteine sulfenate (panel 1). Oxidation of C340 causes the Cys to detach from the Zn-binding core, and a large conformational change occurs in the receptor (panel 2). This structural rearrangement deactivates the receptor so that it no longer stimulates CheA autophosphorylation in the chemotaxis signaling complex (panel 3). Decreasing the cellular pool of CheA-Pi leads to increased smooth swimming and chemoattraction to the HOCl source. CheA, chemotaxis protein A; CheA-Pi, phosphorylated chemotaxis protein A; CheW, chemotaxis protein W; CZB, chemoreceptor zinc-binding; TlpD, transducer-like protein; WT, wild type.

Although the measurement of swimming reversal rates in the presence of a chemoeffector provides useful insight into how bacterial swimming is altered, it is a proxy for the cellular levels of CheA-Pi and does not directly assay chemoattraction in terms of localization of bacteria toward a point source. HOCl degrades at room temperature and is reactive with a variety of organic molecules, so commonly used point-source chemotaxis experiments such as capillaries and soft agar plates were not feasible. Instead, we utilized an assay in which a highly calibrated micropipette can continuously deliver fresh treatment solution to establish a microgradient [21,52]. We tested wild-type *H*. *pylori* strains PMSS1 and G27 for HOCl chemoattraction and indeed found a rapid increase in the bacteria localization to a point source of 5 to 10 mM HOCl, with the bacteria visible in frame after 30 seconds of treatment increasing by approximately 100%; the response was reproducible in experiments performed from different cultures on different days, although the magnitude of the responses showed some variation (Fig 6A, S8 and S9 Figs, and S10 and S11 Movies). The rate and magnitude of this attraction was similar to control experiments performed with urea, and HOCl chemoattraction was eliminated for a *cheA* mutant (S8D Fig and S15 Movie). To test the reversibility of the HOCl attraction, the HOCl point source was removed after establishing an attraction response and the bacteria were observed to disperse (S9 Fig and S12 Movie). HOCl chemoattraction was eliminated for a *tlpD* G27 mutant, indicating that TlpD is required for HOCl sensing (Fig 6C and 6D, S10 Fig, and S13 Movie). A weaker chemoattraction was retained for a *tlpABC* G27 mutant with an increase in bacteria of approximately 70% over buffer after 40 seconds, suggesting TlpD is required and sufficient for HOCl chemoattraction (Fig 6C and 6E, S10 Fig, and S14 Movie).

## Discussion

We report the unexpected discovery that the prevalent gastric pathogen *H*. *pylori*, a causative agent of stomach cancer, is attracted to sources of HOCl by virtue of its cytosolic chemoreceptor TlpD. By reconstituting the chemotaxis complex in vitro, we determined TlpD contains a conserved C340 required for promoting the autophosphorylation of CheA (Figs 1–3). Biochemical analyses revealed that, in TlpD, C340 is tuned to be highly and preferentially reactive toward HOCl and that oxidation of C340 by HOCl stimulates a conformational change in the receptor that presumably inhibits CheA activity, leading to a decrease in the cellular pool of CheA-Pi (Fig 4). TlpD regulation of CheA activity in vitro shows half-maximal inhibition at 110 μM HOCl, and our data suggest this sensing is cooperative at the level of the TlpD homodimer (Fig 4). HOCl treatment of *H*. *pylori* seems to oxidize only a select number of protein thiols, including TlpD (Fig 5B). We surmise that through signal amplification by the formation of TlpD-CheW-CheA sensory arrays, oxidation of just a few TlpD receptors in vivo can inactivate much of the available CheA kinase and thus the cytosol need not reach HOCl concentrations high enough to oxidize all of the TlpD pool to elicit an attraction response. In chemotaxis experiments, *H*. *pylori* exposed to HOCl decrease their rates of swimming reversals to that observed with the well-characterized chemoattractant urea (Fig 5D), they respond to concentrations as low as 250 μM HOCl (Fig 5D–5F), and they rapidly localize toward a point source of HOCl (Fig 6). HOCl attraction was TlpD-dependent and retained in two *H*. *pylori* strains isolated from distant geographic locations (Fig 6).

### Cys340 participates in a Cys-Zn switch with exquisite sensitivity and specificity for HOCl

The molecular basis for TlpD’s specific responsiveness to HOCl is the preferential reactivity of Cys340 with HOCl versus the chemically similar H_2_O_2_. Such specificity can be understood based on differences between the reactivity of these species with protein residues. H_2_O_2_ in the micromolar range almost exclusively reacts with cysteine, and to a lesser extent with methionine, whereas the targets of HOCl are broader and exhibit extremely fast reaction rates; a detailed kinetic analysis measured the reaction rate of HOCl with Cys to be 3.0 × 10^7^ M^−1^ s^−1^ and 3.8 × 10^7^ M^−1^ s^−1^ for Met [53]. That same study showed cysteines bound to zinc are by far the most reactive protein residue with HOCl, achieving a near diffusion-limited rate of 9.3 × 10^8^ M^−1^ s^−1^, almost 3 orders of magnitude greater than cysteine alone [53], and in fact also exhibit decreased reactivity with hydrogen peroxide and oxygen [54]. Previous work has identified numerous other proteins containing zinc-bound cysteines that are targets for HOCl oxidation including Hsp33 [53] and alcohol dehydrogenase, which is 3,000-fold more reactive with HOCl than peroxide [55]. The example we found to be most similar to TlpD is the activation of human neutrophil collagenase that occurs by HOCl oxidation of a single zinc-bound Cys, a mechanism coined as a “Cys-Zn switch” [56,57]. Together, our data indicate the molecular function of TlpD is modulated by reaction of HOCl with C340 to switch between 2 conformations: (1) a “reduced” and Cys-zinc-bound state that is fully folded and activates CheA, and (2) an “oxidized” cysteine-sulfenate state with a partially unfolded CZB domain that does not activate CheA and that can be reduced and resurrected by cellular reductants (Fig 6F).

### HOCl is a prevalent host-produced oxidant encountered by *H*. *pylori*

HOCl, which is produced by the granulocyte-specific enzyme myeloperoxidase from H_2_O_2_ and chloride anion, reaches levels of up to 5 mM at sites of inflammation [49,58,59]. In contrast, maximal gastric concentrations of extracellular H_2_O_2_ are thought to be 1 to 15 μM and perhaps rarely reach approximately 100 μM in the presence of oxidants from dietary sources such as coffee [28,29]. The concentration of superoxide is approximately 2 μM, and it decomposes rapidly, with a half-life on the order of milliseconds [60]. Previous analyses of *H*. *pylori* oxidant sensing [26,27] used concentrations of H_2_O_2_ and superoxide at a much higher concentration than what occurs in vivo. Additionally, although triggered human neutrophils produce several ROS during inflammation, including H_2_O_2_ and superoxide, it seems these are all funneled into the production of HOCl as the major product, because quantitative analyses show essentially all H_2_O_2_ generated is converted to HOCl [49,61].

HOCl is a potent disinfectant that can kill bacteria at micromolar concentrations but is also sufficiently cell permeable to oxidize intracellular thiols at sublethal doses [59,62]. In addition to examples of cytosolic proteins that are targets for HOCl oxidiation discussed above, such as Hsp33 and alcohol dehydrogenase, the bacterial transcription factor NemR within the cytosol uses a reactive Cys residue to selectively sense HOCl to trigger expression of antioxidant enzymes [63]. We showed that *H*. *pylori* is exceptionally resistant to HOCl, likely due to its strong antioxidant defenses including three peroxiredoxins, and high expression of its catalase, KatA [64]. KatA in particular has been shown to be key for *H*. *pylori* to survive ROS bursts from triggered immune cells, and evidence indicates this resilience leads to the chronic release of ROS that damages gastric tissue and results in disease [3]. Our finding that *H*. *pylori* is attracted to sources of HOCl by TlpD suggests that *H*. *pylori* does not just tolerate but can actively seek out sources of HOCl, which would be produced by neutrophils at sites of inflammation. Neutrophils are a hallmark of *H*. *pylori* infection and are actively recruited in response to *H*. *pylori* induction of interleukin-8 [65]. Neutrophils concentrate near the stem precursor and cell zones of the gastric gland epithelium [66], which undergoes proliferation in response to *H*. *pylori* gastric gland colonization [67]. *H*. *pylori* colonization of the gastric glands is an important strategy for the bacteria’s persistent infection of the stomach [68] and requires chemotaxis [52,69], and therefore chemoattraction to HOCl may constitute a strategy *H*. *pylori* uses to persist in neutrophil-infiltrated gastric glands.

This behavior is consistent with other proinflammatory strategies of *H*. *pylori*, such as its degradation of host-secreted extracellular antioxidant glutathione [70], and the fact that its colonization levels are reduced in mice treated with the ROS scavenger ursodeoxycholic acid [71] or sulforaphane, which stimulates host antioxidant protein production through Nuclear factor erythroid 2-related factor 2 [72]. In this regard, *H*. *pylori* belongs to a growing list of bacterial pathogens and pathobionts that can promote and benefit from host inflammation, thereby setting up positive feedback loops that can drive chronic inflammation [73,74]. For example, *Salmonella*-induced inflammation increases the concentrations of colonic tetrathionate and nitrate, which it recognizes as chemoattractants, and it can use these inflammation products as electron acceptors in anaerobic respiration to outcompete fermentation-restricted members of the native microbiota [75–78]. Additionally, other molecular oxidants not tested in this study could be sensed as chemoeffectors by TlpD through a similar mechanism, such as aldehydes and quinones, which are also known to be reactive toward zinc-bound cysteines [79,80], and might provide an explanation for the reported role of TlpD in energy taxis [30,81].

### Interpreting conflicting data of *H*. *pylori* responses to ROS

In contrast to our finding of *H*. *pylori*’s TlpD-dependent chemoattraction to HOCl, previous studies have proposed that TlpD senses ROS as chemorepellents [27,81–83]. Differences both in experimental methodologies and interpretations of results likely contribute to these divergent conclusions. First, we note that measuring *H*. *pylori* chemotaxis can be challenging and often relies on indirect measurements of swimming behavior, such as reversal rates in a uniform chemical solution. Our data indicates that reversal rates are highly variable within a single population of *H*. *pylori* (Fig 5C–5F), which could contribute to inconsistent results using this assay (e.g., opposite reported responses of *H*. *pylori* to paraquat [25, 26]). We found that greater than 100 tracks were required for the mean reversal rate to be well defined (S7C Fig). To better understand *H*. *pylori* responses to ROS, we have focused on ROS species at concentrations reported in the gastric environment. We confirmed our findings of TlpD-dependent, HOCl-induced smooth swimming with a point-source chemotaxis assay that demonstrated localization of *H*. *pylori* toward HOCl (Fig 6, S8–S10 Figs, and S11–S14 Movies).

Secondly, we note the challenges of making inferences about a gene’s function based on the pleiotropic phenotypes of mutant bacteria lacking that gene. *tlpD*-deficient strains have reported colonization defects in mice and gerbils [9,10,81,82], defective chemotactic responses to pH and ROS [9,26,27], and growth defects in the presence of ROS [81]. The mechanistic basis for the colonization defects could thus be due to any combination of the mutant strain’s altered chemotaxis to multiple chemicals in the host environment and its increased sensitivity to ROS. For example, the finding that *tlpD*-mutant strains colonize wild-type mice and gerbils poorly could be alternatively interpreted as evidence of the mutant’s failure to avoid toxic ROS, its failure to establish persistent infection of HOCl-rich gastric glands, or its inherent sensitivity to ROS.

### *H*. *pylori* integrates multiple chemical cues to persist in the gastric environment it shapes

*H*. *pylori* is a notoriously persistent colonizer, often retained for an individual’s lifetime, and previous work highlights the bacteria’s ability to adaptively modify the stomach environment [84–87]. Interestingly, some of the chemoeffectors sensed by *H*. *pylori*’s limited chemoreceptor repertoire reflect the environment of the *H*. *pylori*-infected, as opposed to a naïve stomach, such as low concentrations of urea [21] and presence of autoinducer-2 [88]. Now, we add to this picture chemoattraction to HOCl produced by *H*. *pylori*-induced neutrophil infiltration, a hallmark of the *H*. *pylori*-infected stomach. TlpD seems to be especially important for colonizing the stomach antrum, which has been described in some work as the area of highest inflammation [9,10]. Decreasing host epithelial hydrogen peroxide and immune cell superoxide, which are both precursors for HOCl production, shifts the wild-type *H*. *pylori* infection away from the antrum toward the corpus [83], which could reflect the loss of HOCl as a chemoattractant for locating inflamed regions of the stomach. Clearly, additional studies are needed to elucidate the importance of HOCl sensing for *H*. *pylori* biology and infection. More generally, CZB domain containing proteins are encoded by the genomes of numerous host-associated bacterial species [25], suggesting the broad utility of HOCl sensing for bacteria living together with animals that produce HOCl as an antimicrobial response.

## Materials and methods

### Contact for reagent and resource sharing

Further information and requests for resources and reagents should be directed to and will be fulfilled by the lead contact, Dr. Arden Perkins (ardenp@uoregon.edu).

### Experimental model and subject details

#### *H*. *pylori* growth and preparation

The high motility strain *H*. *pylori* G27 was used for most in vivo assays, and all mutants were created in this background previously [26] and supplied as a gift from Dr. Karen Ottemann (UC Santa Cruz). As an additional control, strain PMSS1 was used in microgradient point-source assays (Dr. Manuel Amieva, Stanford). For growth, frozen stocks were used to inoculate blood plates containing 5% defribrinated horse blood (Hemostat Laboratories, Dixon, CA), 4.4% w/v Columbia agar, 0.02 mg/ml β-cyclodextrin, 0.016 mg/ml amphotericin B, and 0.02 mg/ml vancomycin. Bacteria were grown on plates for 72 hours at 37° C, 10% CO_2_, before passaging to a fresh blood plate and allowed to grow an additional 24 hours. For motility assays, scrapes from blood plates were used to inoculate 3 ml of brucella broth supplemented with 10% FBS and 10 μg/ml vancomycin (BB_10_) for 6 hours at 37°C and 10% CO_2_ shaking at 200 rpm. The liquid cultures were then removed from the shaker and allowed to incubate without shaking for another 1 to 3 hours to obtain maximum motility. Prior to motility and chemotaxis assays, the cultures were diluted with fresh BB_10_ to approximately 0.1 O.D.

#### *E*. *coli* growth and preparation

*E*. *coli* strain UU1250, which is engineered to lack all chemoreceptors and is commonly used in chemotaxis studies [89], was supplied as a gift from Dr. Sandy Parkinson (University of Utah). Frozen stocks of *E*. *coli* were used to inoculate 25 ml of sterile LB media and was grown shaking overnight at 37°C. In the morning, 100 μL of the overnight culture was diluted into 5 ml of fresh TB media and allowed to grow until reaching an O.D. of approximately 0.5 and then was diluted to approximately 0.1 O.D. for use in motility assays.

### Method details

#### Sequence analysis and conservation of TlpD

The full-length protein sequence of *H*. *pylori* TlpD from strain SS1 (Uniprot entry A0A1U9IUC7) was used for BLAST [31] searches of the nonredundant sequence database using default threshold values. We performed one search for only *H*. *pylori* sequences and a second search excluding *H*. *pylori* sequences. We manually curated these sequences to retain only those that were chemoreceptors and also contained a C-terminal CZB domain. Of these, some sequences lacked the N-terminal region corresponding to approximately the first 120 residues; because we were interested in analyzing the conservation of TlpD cysteine positions, and three of those positions were contained in the N-terminal region, we imposed an additional restriction that we only included sequences in the analysis that lacked no more than the first 30 N-terminal residues. This resulted in a total of 459 putative TlpD homologues. We performed a multisequence alignment of this subset using Clustal Omega [90] and generated a relatedness tree using PhyML [91] (Fig 1C).

To create the homology model of the TlpD monomer, we submitted the *Hp*TlpD (SS1 strain) sequence to the i-Tasser server [32] as two separate pieces corresponding to the N-terminal region (residues 1–120) and the coiled-coil and CZB domain (residues 121–433), and the subsequent predicted models were manually joined (Fig 1D). Although generation of the model could be guided by crystal structures of the chemoreceptor coiled-coil region and a CZB domain from *E*. *coli* DgcZ, the TlpD N-terminal region is unlike any experimentally determined structure, and so the fold of this region remains highly speculative. We performed a sequence conservation analysis at the five cysteine positions found in *Hp*TlpD (C35, C103, C117, C308, and C340) for *H*. *pylori* sequences and *nonpylori* sequences using the WebLogo server [19] and mapped these onto the i-Tasser homology model (Fig 1D and 1E).

#### Purification of recombinant proteins

pBH plasmids with ampicillin resistance and a T7 promotor system [20] were ordered from GeneScript that contained the sequences for *H*. *pylori* TlpD (SS1 strain, Uniprot: A0A1U9IUC7) and C340A mutant, CheW (SS1 strain, Uniprot: A0A1U9ITT8), CheA (G27 strain, Uniprot: B5Z859), and *S*. *enterica* typhimurium McpA (LT2 strain, Uniprot: Q8ZM22) each with the N-terminal His tag: MGHHHHHHDYDIPTTENLYFQGS. For these constructs, ArcticExpress DE3 competent *E*. *coli* cells (Agilent) were transformed with plasmids by heat shock and plated on +ampicillin LB agar plates. After overnight growth at 37°C, single colonies were selected for growth and large scale protein expression. Briefly, individual colonies were used to inoculate 25 ml LB/+AMP cultures and grown overnight shaking at 37°C. The following morning, 5 ml of overnight culture was added to 4 × 1 L cultures of LB/+Amp and allowed to grow shaking at 37°C to O.D. 0.6 to 0.8. Cultures were then transferred to a temperature-controlled incubator and allowed to grow shaking for 30 minutes at 10°C, prior to induction with 1 mM IPTG and growth overnight at 10°C. After approximately 16 hours, cultures were harvested by centrifugation at 5,000 rpm at 4°C. Similar protocols were used for expression of the CZB domain of *E*. *coli* DgcZ (DgcZ-CZB in strain K12), which was supplied as a gift from Dr. Tilman Schirmer of University of Basel [18], using expression with Rosetta cells and a pet19b vector.

For protein purification, cells were diluted into ice-cold lysis buffer containing 10 mM imidazole, 50 mM HEPES, 10% glycerol, 300 mM NaCl, and 0.5 mM TCEP (pH 7.9). Cells were kept on ice and lysed by sonication and then centrifuged at 15,000 rpm to separate out the insoluble fraction. The soluble portion was retained and applied to a prepacked gravity column of Ni-NTA agarose beads (Qiagen) equilibrated with lysis buffer. Lysate was incubated with the beads for 30 to 60 minutes and allowed to flow through the column over the beads twice. The column was then washed with lysis buffer to remove contaminates until no further protein was observed in the flow through, as monitored using a Bradford assay. Protein was eluted by the addition of lysis buffer containing 300 mM imidazole. To obtain TlpD lacking the N-terminal His tag, TlpD(-His), the eluted protein fractions were dialyzed into lysis buffer and then incubated with 1 mg TEV protease per 20 ml elution volume overnight (New England Biolabs) and repurified. Elution fractions were judged for purity by SDS-PAGE and pooled before further purification by gel filtration on an Akta FPLC. All proteins were obtained in high yields on the order of 40 to 100 mg per prep, and fractions containing pure protein were dialyzed into appropriate buffers and concentrated; TlpD, TlpD(-His), TlpD-C340A, and CheA were found to be highly soluble and stable up to 300 μM, but CheW could only be concentrated to approximately 150 μM without precipitation. Attempts at removing the N-terminal His tag of CheW were unsuccessful and caused the protein to precipitate. Proteins were flash frozen in liquid nitrogen and stored at −80°C.

#### Preparation of bleach solutions

High grade sodium hypochlorite (NaOCl) was obtained as a 10% to 15% solution (Sigma-Aldrich, St. Louis, MO). Precise concentration of NaOCl was assayed directly using the extinction coefficient of 350 M^−1^ cm^−1^ measured at 293 nm in water [92]. NaOCl stock was kept cold and in the dark until needed, and bleach solutions for assays were made fresh. Concentrated solutions of NaOCl are extremely basic, and so in all cases, the preparation of NaOCl stock solutions (1–100 mM) was measured and adjusted by addition of HCl to pH 7 and kept in PBS buffer (pH 7) prior to experiments. The solution pH was monitored by a Mettler Toledo pH probe (ThermoScientific, Eugene, OR) and use of pH indicator strips (Millipore, St. Loius, MO). The pKa of HOCl is 7.53, and so the experiments performed in this study contain a mixture of hypochlorous acid (HOCl) and hypochlorite (^−^OCl) in solution, but for simplicity, we refer to these solutions simply as HOCl.

#### ATP radio-labeling and functional assays of reconstituted chemotaxis signaling complex

Most functional assays were performed at 20°C in a standard kinase buffer containing 50 mM Tris (pH 7.5), 100 mM NaCl, and 10 mM MgCl_2_, with typical reactions containing purified chemotaxis proteins in approximately stoichiometric concentrations: 4 μM CheA, 8 μM CheW, and 24 μM TlpD dialyzed into kinase buffer. ATP solutions were prepared with a 500:1 ratio of cold ATP (Thermofisher, Eugene, OR) to hot ATP [ɣ-32P] (Perkin Elmer, Waltham, MA) diluted into kinase buffer. Time courses were carried out in a 96-well clear bottom plate (Corning) with reactions started by the addition of ATP. At each time point, 3 to 10 μl samples were extracted and immediately quenched in a solution of 190 to 197 μL of 75 mM H_3_PO_4_ and 1 M NaCl. Subsequently, samples were added to a Whatman Minifold 96-well slot blotter and drawn through a HyBond PVDF nitrocellulose membrane by vacuum. Three additional washes with 200 μL quenching solution were performed to remove excess ATP. The nitrocellulose was dried and then imaged using a phosphoimaging cassette (Molecular Dynamics, Berkeley, CA) with a 30 to 60 minute exposure and then scanned using a Storm 825 imager (GE Healthcare, Chicago, IL). Data were quantified by densitometry using Image Studio. Identically sized circles were used for all measurements, and raw values from experimental samples were normalized against “time zero” samples that contained only ATP and no kinase.

For testing putative chemoeffectors, treatments of HOCl, H_2_O_2_, paraquat, TCEP, and zinc acetate were added to proteins 1 hour prior to the start of the reaction (Fig 3B and 3D, and S2C Fig and S2F Fig). Preoxidized TlpD was prepared by treatment of TlpD alone with 250 μM HOCl for 30 minutes, then quenched with 1 mM methionine prior to addition of CheW, CheA, and ATP (Fig 3C). We performed similar experiments using wild-type TlpD, CheW, and CheA across a variety of pHs to test whether TlpD may be involved in directly sensing pH. TlpD has been implicated to be involved in chemorepulsion from acid and chemoattraction to alkaline pH [9], and if this sensing was direct, we expect in the reconstituted chemotaxis complex assay this would be reflected as higher CheA activity at more acidic pH, leading to more swimming reversals and chemorepulsion and less CheA activity at basic pH. Our results actually found the opposite to be true, because CheA autophosphorylation activity increased at more alkaline pH (S2E Fig). This has been previously reported for studies with *E*. *coli* CheA alone [93], suggesting the increase in activity may be due to the CheA autophosphorylation reaction being more favored at alkaline pH, although further work is needed to confirm this.

#### Detection of cysteine sulfenic acid by western blotting

Experiments measuring CZB oxidation were prepared with either 20 μM TlpD, TlpD-C340A, *E*. *coli* DgcZ-CZB, or *Salmonella* McpA in PBS buffer (pH 7) with 10 mM freshly prepared dimedone (Sigma). The proteins were treated with various concentrations of HOCl for 1 hour, and then 5 μl of sample was quenched into 195 μl of quenching buffer (see above) and drawn through a HyBond PVDF nitrocellulose membrane by vacuum. The membrane was washed 3 times with quenching buffer and then once with TBST buffer (50 mM Tris [pH 7.5], 150 mM NaCl, 0.1% Tween-20). Rabbit α-cysteine sulfenic acid primary antibody (Kerafast, Boston, MA) was used to detect dimedone-modified cysteines [45] using a 1:4,000 dilution in a blocking buffer of 5% milk in TBST. The membrane was incubated with primary antibody overnight with gentle rocking. The following day, the membrane was washed three times with 20 ml of TBST for 15 minutes, followed by incubation with α-rabbit-HRP secondary antibody (Santa Cruz Biotechnology, Santa Cruz, CA) at a 1:4,000 dilution in blocking buffer for 1 hour. The membrane was again washed three times with 20 ml of TBST for 15 minutes, and then a Pierce ECL detection kit was used to visualize the blot using chemiluminescence.

For detecting formation of cysteine sulfenic acid in *H*. *pylori* cells in response to HOCl treatment, *H*. *pylori* G27 wild type and *tlpD* mutant were grown overnight in BB_10_ media and diluted to be O.D. 1.0. Cells were treated with dimedone and then either PBS buffer (pH 7) or 1 mM HOCl diluted into PBS buffer (pH 7) for 30 seconds, and then quenched by 2 mM methionine for 5 minutes, followed by addition of SDS to lyse the cells. Samples were then run on SDS-PAGE gels, blotted, and probed with rabbit α-Tlp primary antibody at a 1:4,000 dilution (K. Ottemann), stripped with OneMinute Western Blot Stripping Buffer (GM Biosciences, Frederick, MD) and reprobed with α-cysteine-sulfenate primary antibody (green). Blots were visualized using chemiluminescence, as described above. Full images of blots are shown in S5 Fig and S6 Fig.

### Mass spectrometry

20 μM of pure recombinant TlpD in PBS buffer (pH 7) was prepared for analysis by treatment with 1 mM TCEP, H_2_O_2_, or HOCl for 1 hour. Subsequently, the samples were alkylated by the addition of 5 mM iodoacetamide and allowed to react for 30 minutes at room temperature sheltered from light. The sample was then buffer-exchanged with fresh buffer three times by centrifugation using a 10 kDa cutoff and flash frozen in liquid nitrogen prior to analysis. Mass spectrometric analysis of TlpD was performed as a service by Dr. Larry David at Oregon Health and Science University’s (OHSU) Proteomics Shared Resource Facility. As per their request, we include the following statement: “Mass spectrometric analysis was performed by the OHSU Proteomics Shared Resource with partial support from NIH core grants P30EY010572, P30CA069533, and S10OD012246.” Protein samples were subjected to digestion with LysC, and 3 μg of digest was analyzed on a Q-Exactive HF instrument using a 75 μm × 250 mm nanospray C18 column. MS/MS data were searched with Sequest against an *E*. *coli* database supplemented with the TlpD protein sequence. False discovery was controlled using a reversed sequence database and only q-scores below 0.05 were accepted. C340 was located within the peptide NCRLGKWYYEGAGK (567.94415 Da), and carbamidomethylated (+57.02146 Da), sulfinate (+31.98983 Da), and sulfonate (+47.98474 Da) forms were detected. Ion extractions were performed for the +3 charge states for the 3 forms of this peptide using a 2 ppm tolerance, and peaks were integrated to obtain absolute values for quantification and comparison of modifications across experiments (Fig 4E and S3C–S3E Fig).

### Fluorescence, CD spectroscopy, and analytical ultracentrifugation

For Zn-chelation assays, we used the fluorescent probe Zinpyr-1 (Abcam, Eugene, OR) with excitation at 492 nm and maximum emission near 527 nm; 5 mM stock solutions of Zinpyr-1 were prepared in DMSO and diluted to 50 μM final concentration for experiments with zinc controls and TlpD in a buffer of 100 mM Tris (pH 7) and 300 mM NaCl. Samples were loaded into a 2 × 2 mm quartz cuvette (Starna Cells Inc. Atascadero, CA) and fluorescence was monitored at 20°C using a FluoroMax-3 Spectrofluorometer (HORIBA Scientific, Austin, TX). For time courses, fluorescence was measured every 60 seconds over the course of 3 hours. Identical samples containing only buffer were used as blanks, and final spectra shown are blank-subtracted spectra, but these were minimally different (S2 Fig).

For intrinsic protein fluorescence assays with TlpD and the C340A mutant, we used an excitation wavelength of 295 nm, to optimize signal from the single Trp345, and monitored emission in the 300 to 400 nm range (Fig 4G). Samples were prepared in a buffer of 25 mM NaCl and 20 mM Tris (pH 7), and experiments were conducted at 20°C. Samples were pretreated for 1 hour prior to measuring fluorescence for endpoint fluorescence spectra of TlpD HOCl treatments (Fig 4G–4I), or subsequently treated with TCEP for 30 minutes (Fig 4J). Fluorescence anisotropy experiments for recombinant TlpD in PBS buffer (pH 7) and 1 mM TCEP were collected with excitation at 295 nm and monitoring emission at 340 nm (Fig 2B). Intrinsic fluorescence was too weak at low protein concentrations to be reliably detected, and so a K_D_ value could not be calculated from these data but were still useful to indicate that the protein is maximally oligomerized by approximately 16 μM, consistent with monomer/dimer ratios predicted from AUC experiments (Fig 2B and S1 Fig)

Protein CD was performed for TlpD with a Jasco J-810 spectropolarimeter using a 1 mm path length quartz cuvette. All spectra for fluorescence and CD experiments were corrected for background by subtracting signal from buffer-only samples. To obtain a melting and cooling curve, 50 μM TlpD in a buffer of 150 mM NaCl and 17.5 mM sodium citrate (pH 7) was heated from 25°C to 95°C in 0.5°C increments per 5 minutes and then cooled back to 25°C (S2D Fig). For HOCl titration and unfolding experiments, 5 μM TlpD was treated with various concentrations of HOCl for 10 minutes in a buffer of PBS (pH 7), then desalted with a Micro Bio-Spin Column (Bio-Rad) into a buffer of 150 mM NaCl and 17.5 mM sodium citrate (pH 7), and then analyzed by CD at 20°C (S3B Fig).

Analytical ultracentrifugation experiments were performed for recombinant TlpD and TlpD(-His) using a Beckman Proteome Lab XL-I centrifuge and a 60 Ti 4-cell rotor. Interference experiments were performed using 1, 2, and 3 × 10 μM TlpD and 3 × 28 μM TlpD(-His) in PBS (pH 7) and 1 mM TCEP at 20°C, with a total volume of 400 μl at 55,000 rpm. Data were processed with SedFit [94] using a continuous distribution model with rmsd of the final data sets <0.016 (Fig 2B and S1 Table).

#### Bacterial motility velocity assays

Bacteria were grown as described above and diluted in fresh media to be approximately O.D. 0.1. For treatments, 2 μl of motile bacteria combined with 2 μL of chemotaxis buffer (10 mM PBS [pH 7] and 1 mM EDTA) or HOCl diluted into chemotaxis buffer from a concentrated stock of HOCl (pH 7). The samples were mixed by gentle pipetting, applied to a 10-well slide (MP Biomedicals, Eugene, OR), covered with a 22 mm micro cover glass (VWR), and visualized immediately. For each experiment, brightfield videos of swimming bacteria were recorded using a Nikon Eclipse Ti inverted scope using a 20× objective and equipped with an AirTherm temperature-controlled sample chamber set to 37°C. Videos were 30 seconds in duration and recorded at 25 frames per second (Fig 5A). The number of bacteria tracks counted for each HOCl concentration were as follows: *E*. *coli*: buffer (409), 500 μM (377), 1 mM (283), 2 mM (324), and 3 mM (no motile bacteria detected); *H*. *pylori* buffer (91), 500 μM (39), 1 mM (33), 5 mM (70), 6 mM (60), 7 mM (23), and 8 mM (no motile bacteria detected).

#### *H*. *pylori* chemotaxis assays

*H*. *pylori* were grown as described above and diluted in BB_10_ to be approximately O.D. 0.1 and checked for motility after 30 to 60 minutes; the bacteria were used for motility and chemotaxis assays once the culture was observed to be near 100% motile. For experiments monitoring changes in reversal rates upon chemoeffector treatment, 1 μl of motile cells were added to 1 μl of 2× chemoeffector treatment solution, mixed gently by pipetting, and imaged identically as described above for motility velocity experiments. For treatments with acid, a 60 mM HCl stock diluted in BB_10_ was added to cells, resulting in lowering of the total solution pH to about 4.5. All other chemoeffector treatments were made from concentrated chemoeffector stocks that were made fresh in a buffer of PBS (130 mM NaCl, 3 mM KCl, 10 mM Na_2_HPO_4_, and 2 mM KH_2_PO_4_ [pH 7]; Fig 5C–5E and S1–S9 Movies). The pH of stock chemoeffector solutions were adjusted to be pH 7 and further diluted in PBS to obtain 2× solutions for experiments. Three separate experiments were performed for each treatment, and the resulting data are reported in Fig 5D–5F and S7B Fig.

Point-source micropipette assays were performed as previously described by Huang and colleagues [21]. An Eppendorf Femtotip II microinjection micropipette, loaded with either a buffer of 500 mM sodium phosphate (pH 6.7), 5 mM urea diluted in buffer, or 5 to 10 mM HOCl diluted in buffer, was inserted into a swarm of motile *H*. *pylori* using an Eppendorf micromanipulator. The pH of treatment solutions was measured prior to experiments to ensure addition of chemoeffectors did not shift solution pH. Treatment was initiated by applying a pressure of 30 hPa to maintain a constant flow of 0.372 pl per minute, which is low enough to not physically perturb the swimming bacteria [21]. Videos of swimming bacteria were captured at 30 frames per second on a Zeiss Axiovert-35 inverted microscope with a 40× objective and experiment temperature was kept stable at 37°C with a heated stage (Fig 6A–6E, S8–S10 Figs, and S10–S15 Movies). For each experiment, a baseline for the number of bacteria in the field of view was established pretreatment, and then the micropipette was lowered into the swarm, and a gentle flow of treatment solution was initiated. Additional experiments were performed in which the point source was removed after establishing an attraction response to monitor the rate of dispersal (S9 Fig and S12 Movie). Note that the point source solution is purposefully concentrated in order to establish a microgradient (Fig 6) and that this differs from experiments monitoring reversal rates and motility assays in which the bacteria are completely immersed in a solution of chemoeffector at a given concentration (Fig 5). Therefore, although we report that immersion of *H*. *pylori* cells in solutions containing >7 mM HOCl causes loss of motility, bacterial swimming is uninhibited by the point source of 10 mM HOCl and are able to leave and return without apparent differences in motility.

### Quantification and statistical analysis

#### Curve fitting of reactions and calculation of thermodynamic constants

Radio-ATP time course assays were run to completion (15 minutes to 2 hours depending on the experiment), and raw data were normalized with the assumption that endpoint values reflected maximally phosphorylation CheA. For Michaelis-Menten assays using CheA and varying [ATP], the data were fit assuming the second-order reaction proceeds as CheA + ATP ↔ CheA-Pi + ADP so that the radioactivity measured is directly proportional to [CheA-Pi], and [CheA] and [ATP] can be deduced for any time point by knowing initial concentrations. Thus, experimental data were fit to the second-order rate equation

$$k_{obs}=\frac{\frac{1}{\left[CheA_{0}]-[ATP_{0}\right]}*(\ln\left(\frac{\left[CheA_{t}][ATP_{0}\right]}{\left[ATP_{t}][CheA_{0}\right]}\right))}{t}$$ (1)

where [CheA_0_] is the starting concentration of CheA, [ATP_0_] is the starting concentration of ATP, [CheA_t_] is the concentration of CheA measured at time *t*, and [ATP_t_] is the concentration of ATP measured at time *t*. A least-squares fit of the mean data values to Eq 1 were performed using Microsoft Excel’s Solver addon to minimize the error of the fit with the data values weighted by the standard deviation of each sample, calculated as

$$\Sigma \left(R=\frac{{\left(obs-calc\right)}^{2}}{std}\right)$$ (2)

where R is error, *obs* is the measured value of [CheA], *calc* is the calculated concentration of [CheA] based on Eq 1, and *std* is the standard deviation of the sample measurements. Individual k_obs_ values from time courses were plotted as a function of ATP concentration, with error calculated as in Eq 2, and fit to the Michaelis-Menten equation (Fig 2A)

$$k=\frac{Vmax*[ATP]}{K_{m}+[ATP]}$$ (3)

where *k* is the reaction rate, *V*_*max*_ is maximum velocity, *K*_*m*_ is the substrate concentration required for half-maximal velocity, and [ATP] is the concentration of ATP. This resulted in a calculated K_m_ of CheA (at 4 μM) for ATP of 136 μM, and V_max_ of 0.0033 μM min^−1^. These values were used to calculate k_cat_ using the equation

$$k_{cat}=\frac{\left[CheA_{0}][ATP_{0}\right]}{K_{m}+[ATP_{0}]}$$ (4)

where [CheA_0_] is the concentration of CheA and [ATP_0_] is the initial concentration of ATP. This yielded a k_cat_ of 8.25 × 10^−3^ min^−1^, indicating that CheA autophosphorylation is extremely slow in the absence of activators. Subsequent time course reactions were run under pseudo first-order conditions using 1 mM ATP, and k_obs_ values were calculated from fits of the data as described above (Eq 1) to the pseudo-first-order rate equation

$$\left[CheA-Pi]=[CheA_{0}](1-e^{kt[ATP]}\right)$$ (5)

For estimating kinetically defined K_D_ values for signaling complex components (Fig 2D and 2E), we performed a series of time course reactions titrating a single protein component, either CheW or TlpD, against 4 μM CheA and 1 mM ATP, and all other components were kept at a constant concentration. The data for each time course were fit to Eq 5 to calculate k_obs_, and the series of k_obs_ values were normalized assuming maximal values corresponded to fully bound complex (either CheA-CheW, or CheA-CheW-TlpD). The k_obs_ values were plotted against concentration of the titrated component and fit to a two-component binding (Hill) equation:

$$[AB]=\frac{B_{T}}{K_{D}+B_{T}}$$ (6)

where it is assumed binding occurs as A+B↔AB, [AB] is the concentration of the bound complex, B_T_ is the total added titrated component, and K_D_ is the thermodynamic dissociation constant. Using this method, we estimated the K_D_ of complex formation of CheA+CheW↔CheA-CheW to be 14.6 μM. In the case of the titration of TlpD against CheA and CheW (Fig 2E), we were unable to use a saturating amount of CheW due to the limits of the protein binding capacity of the nitrocellulose membrane. Instead, we used 4 μM CheA and 40 μM CheW and approximated the concentration of the CheA-CheW complex to be 2 μM, based on our previous titration of CheW and CheA, although this equilibrium could be shifted by TlpD addition. However, data were well-fit using this estimate, producing a K_D_ for TlpD to CheA-CheW of 15.2 μM, and test fits using values between 2 and 4 μM CheA-CheW complex only subtly changed the calculated K_D_ by approximately 1 μM. Eq 6 was also used to fit quantified data from fluorescence and western blot experiments using Hill coefficients of 1 and 2 (Fig 4D and 4H).

Calculation of the TlpD and TlpD(-His) dimerization K_D_ was performed by integrating the monomer and dimer peaks from individual analytical ultracentrifugation experiments to obtain the monomer/dimer ratio, which directly relates to the K_D_ of a two-species oligomerization assuming that

$$K_{D}=\frac{M^{2}}{D}$$

and

$$P_{0}=M+2D$$

and

$$C=\frac{M}{D}$$

where M is the concentration of monomer, D is concentration of dimer, C is the ratio of monomer to dimer, and P_0_ is the total protein concentration; then

$$K_{D}=\frac{C^{2}*P_{0}}{C+2}$$ (7)

#### Tracking of bacterial swimming

We used in-house MATLAB-based automated particle tracking software from the laboratory of Dr. Raghuveer Pathasarathy (University of Oregon) to track bacteria swimming paths [51]. This software is freely available and can be downloaded at https://pages.uoregon.edu/raghu/particle_tracking.html. Videos of bacteria were first converted to a series of tif files using ImageJ [95], inverted so that bacteria appeared as bright spots on a dark background, and then input into the tracking software. A set of standard threshold values specific to the particle tracking software were applied to all video data as follows: “Objects” were identified using bpfilter 3, nsize 13, gradob 0, applying a std. threshold of 3.99; nearest objects between frames within 5 pixels were joined to create tracks of the moving particles. To separate swimming bacteria tracks from other particles, tracks were only retained that were at least 1 second long (monitored over a minimum of 25 frames) and moved greater than 2 std above the mean deviation in position (Fig 5).

#### Quantification of *H*. *pylori* swimming reversals

Bacterial tracks as XY-coordinates from chemotaxis assays were input into Excel to quantify swimming reversals by calculating vector changes along the swimming trajectory. Here, we define a reversal as being a direction change of >300° with positions between frames at least deviating by 0.325 μm to minimize reversals being counted when the bacterium is essentially motionless (S7A Fig). We found these to be reliable thresholds for replicating results based on visual analysis and removed a potentially large source of observer bias and inconsistency by applying the same standards to all samples analyzed. Mean reversal rates and sample distributions are listed in S2 Table.

#### Quantification of *H*. *pylori* chemoattraction to point sources of HOCl

Data from micropipette chemotaxis assays were quantified using the automated tracking software described above (Fig 6A–6E). These measurements were normalized by dividing the number of bacteria detected in each frame by the average number of bacteria observed over the 10 seconds immediately prior to insertion of the micropipette and treatment to obtain a plot of “Bacteria %” change over time (Fig 6B and Fig 6D and 6E). For assays with the *tlpABC* mutant that resulted in less substantial chemoattraction, we performed additional statistical analysis to help determine whether the response was significant (S10C Fig). We fit bacteria counts over 1,200 frames (40 seconds) of post-treatment video from 3 independent replicates to a simple linear model for HOCl treatments. A significant positive correlation of 0.3125 was found for HOCl treatment (*p* ≤ 0.00001) compared with buffer (*p* = 0.997).

See S3 Table for a list of key resources. See S1 Data for data values presented in figures.

## Supporting information

S1 Fig TlpD monomer/dimer equilibrium as a function of protein concentration. Formation of TlpD homodimers in a buffer of PBS (pH 7) and 1 mM TCEP was analyzed in a series of AUC experiments for recombinant TlpD with either an intact N-terminal His tag, TlpD(+His), or with the tag cleaved by TEV protease, TlpD(-His). The TlpD(+His) and TlpD(-His) K_D_ values were found to be 188.5 nM SEM ± 121.4 nM and 64.8 nM SEM ± 2.1 nM, respectively. Relative amounts of monomer (dashed lines) and dimer (solid lines) estimated from these values are shown for TlpD(+His) (black) and TlpD(-His) forms as a function of total protein concentration. TCEP, Tris(2-carboxyethyl)phosphine; TlpD, transducer-like protein D.
(TIF)

S2 Fig Analysis of TlpD Sensing of Zn^++^, pH, and paraquat. (A) A series of functional assays using reconstituted TlpD-CheW-CheA signaling complex with addition of 0 (red), 0.25× (orange), 0.5× (yellow), 1× (lime green), 2× (teal), 4× (cyan), 8× (dark green), and 16× (dark blue) zinc sulfate relative to [TlpD]. Decreases in activity were due to protein precipitation. (B) Shown is a fluorescence emission spectrum (ex. 492 nm) using 50 μM Zinpyr-1 in which the addition of 300 μM zinc acetate exhibits an increase in fluorescence at 547 nm (black dashes), but the addition of 30 μM TlpD for 10 minutess (purple) does not increase fluorescence over Zinpyr-1 alone (black, closely overlays with purple). (C) Time courses monitoring fluorescence (ex. 429/em. 527 nm) for 50 μM of the Zn-chelating probe Zinpyr-1 with a positive control of 50 μM zinc acetate (black dashes),versus 50 μM TlpD (black solid) or 50 μM heat-denatured TlpD (black dotted). (D) Shown left is a CD spectra of 50 μM TlpD in a buffer of 150 mM NaCl and 17.5 mM sodium citrate (pH 7). Shown right is a melting curve in which the sample was heated from 25°C to 95°C and then cooled back to 25°C with the CD at 210 nm (blue-orange) and 225 nm (blue-red) monitored as a function of temperature. (E) Shown are results from a series of functional assays showing relative rates of CheA autophosphorylation at low (pink), neutral (yellow), and basic (blue) pH. Experiments were run with 1 mM ATP, 4 μM CheA, 8 μM CheW, and 24 μM TlpD with 10 mM MgCl_2_, 100 mM NaCl, and 200 mM of total buffer comprised of a combination of sodium citrate and tris. (F) Functional assays are shown with either 1-hour pretreatment with buffer (red), 10 μM paraquat (light green), or 100 μM paraquat (dark green). CD, circular dichroism; CheA, chemotaxis protein A; CheW, chemotaxis protein W; em., emission; ex., excitation; TlpD, transducer-like protein D.
(TIF)

S3 Fig Effects of HOCl oxidation on TlpD. (A) Data from Fig 4D shown with a logarithmic x-axis. (B) Shown is a titration of HOCl against 5 μM TlpD monitored by CD. Samples were treated at the concentrations indicated for 10 minutes in a buffer of PBS (pH 7) and then desalted into a buffer of 150 mM NaCl and 17.5 mM sodium citrate (pH 7) prior to analysis to reduce voltage and facilitate CD measurement. (C–E) Shown are MS/MS ion extractions for the TlpD C340-containing peptide NCRLGKWYYEGAGK from samples treated with 1 mM TCEP (C), hydrogen peroxide (D), or HOCl (E). For each sample, ion extractions for +3 charged peptides containing modifications corresponding to alkylation are shown on top (C340-SONH_2_, unreacted cysteine thiols modified by iodoacetamide), oxidation of C340 to a cysteine sulfinate (C340-SO_2_^−^) are shown in the middle, and oxidation of C340 to a cysteine sulfonate (C340-SO_3_^−^) are shown on the bottom on a fixed scale. Integrations values for peaks are noted with “AA.” The experiment and analysis are described further in the Method details. CD, circular dichroism; MS/MS, tandem mass spectrometry; TCEP, Tris(2-carboxyethyl)phosphine; TlpD, transducer-like protein D.
(TIF)

S4 Fig Oxidation of CZB domains by HOCl. CZB protein domains from different bacterial species were analyzed for reactivity toward HOCl and formation of cysteine sulfenic acid. Shown are reactions of purified *Hp*TlpD (data from Fig 4D, black line and open circles), *S*. *enterica* McpA (green), and the CZB domain of *E*. *coli* DgcZ (also called YdeH) with various concentrations of HOCl, run as in Fig 4D. Solid lines are fits of the data to the Hill equation with a coefficient of 2, and markers shown are the average of triplicate independent measurements. Error bars are the sample standard deviation. CZB, chemoreceptor zinc-binding; DgcZ, diguanylate cyclase Z; *Hp*TlpD, *Helicobacter pylori* transducer-like protein D; YdeH,protein product of gene ydeH.
(TIF)

S5 Fig Western blot of HOCl-treated WT *H*. *pylori* cells. Shown is a single western blot of independent treatments of *H*. *pylori* G27 cells with PBS buffer (pH 7; lanes 1–4) or 1 mM HOCl in PBS buffer (pH 7; lanes 5–9). The blot was probed with α-Tlp primary antibody (pink), stripped, and reprobed with α-Cys-SOH (green). The resulting chemiluminescence images from each probe are shown overlaid and merged on the bottom. The protein ladder for each image was collected on a separate channel and overlaid with the chemiluminescence data. See Fig 5B and Method details for additional information. WT, wild type.
(TIF)

S6 Fig Western blot of HOCl-treated *tlpD H*. *pylori* cells. Shown as in S5A Fig, single western blot of independent treatments of *H*. *pylori tlpD* G27 cells with PBS buffer (pH 7; lanes 1–4) or 1 mM HOCl in PBS buffer (pH 7; lanes 5–9).
(TIF)

S7 Fig Quantification of reversal rates in *H*. *pylori* swim assays. (A) Shown left is a representative swimming track for wild-type *H*. *pylori* G27 with each dot corresponding to the bacteria’s position every 40 ms. Reversals are highlighted as blue circles. Shown right is the definition of a reversal applied for quantification in this study and in Fig 5. (B) Control experiments with *cheA H*. *pylori* are shown with identical buffer and HOCl treatments as in Fig 5. Data shown are from 3 independent experiments. The baseline of the wild-type reversal rate treated with PBS buffer (pH 7) is indicated as a black dashed line. Black solid lines indicate the mean reversal rate under each condition. Because *cheA* is a chemotaxis-null mutant, these values can be considered as the false-positive rate of a fully smooth-swimming population, in which the swimming trajectory by chance elicits a trajectory change indistinguishable from a chemotactic reversal. (C) Shown is the mean reversal rate for representative data sets versus number of bacteria tracks.
(TIF)

S8 Fig *Helicobacter pylori* attraction to a point source of HOCl. (A–C) Shown are data from representative chemotaxis experiments collected on different days showing the responses of G27 and PMSS1 *H*. *pylori* to a micropipette point source containing a solution of 5 to 10 mM HOCl and 500 mM PBS (pH 6.7). Data shown are relative bacteria counts in the frame of view during pre- and post-treatment for each frame of video (30 fps) normalized to counts prior to treatment. Treatment begins at time 0, indicated with a yellow arrow. (D) Additional controls showing response to the chemoattractant urea and the lack of a response to HOCl for a chemotaxis-null *cheA* mutant.
(TIF)

S9 Fig *H*. *pylori* dispersal following removal of HOCl point source. (A–D) Shown are data from representative point-source chemotaxis assays in which the point source is removed after the accumulation of bacteria in the field of view (indicated by black arrows).
(TIF)

S10 Fig *H*. *pylori tlpD* and *tlpABC* responses to a HOCl point source. Shown are data from point-source chemotaxis assays collected on different days with G27 *tlpD* (A) and G27 *tlpABC* (B–C) mutants with either 500 mM PBS (pH 6.7; buffer) or 10 mM HOCl and 500 mM PBS (pH 6.7) in the micropipette. For clarity, data shown in (C) are smoothed by averaging normalized bacteria counts over 1 second intervals (30 frames). A linear model (black line) is fit to the post–HOCl-treated *tlpABC* counts over all frames, showing a positive correlation of approximately 0.3 over 40 seconds for 3 independent replicates (*n* = 3,600, *p* < 0.00001).
(TIF)

S1 Movie Wild-type *H*. *pylori* G27 treated with PBS buffer (pH 7). (MP4)

S2 Movie Wild-type *H*. *pylori* G27 treated with 1 mM urea in PBS buffer (pH 7). (MP4)

S3 Movie Wild-type *H*. *pylori* G27 treated with 1 mM HOCl in PBS buffer (pH 7). (MP4)

S4 Movie *cheA H*. *pylori* G27 treated with PBS buffer (pH 7). (MP4)

S5 Movie *cheA H*. *pylori* G27 treated with 1 mM HOCl in PBS buffer (pH 7). (MP4)

S6 Movie *tlpD H*. *pylori* G27 treated with PBS buffer (pH 7). (MP4)

S7 Movie *tlpD H*. *pylori* G27 treated with 1 mM HOCl in PBS buffer (pH 7). (MP4)

S8 Movie *tlpABC H*. *pylori* G27 treated with PBS buffer (pH 7). (MP4)

S9 Movie *tlpABC H*. *pylori* G27 treated with 1 mM HOCl in PBS buffer (pH 7). (MP4)

S10 Movie HOCl point-source assay with wild-type *H*. *pylori* G27. Micropipette contains either 500 mM PBS (pH 6.7) or 10 mM HOCl and 500 mM PBS (pH 6.7).
(MP4)

S11 Movie HOCl point-source assay with wild-type *H*. *pylori* PMSS1. Micropipette contains either 500 mM PBS (pH 6.7) or 10 mM HOCl and 500 mM PBS (pH 6.7).
(MP4)

S12 Movie Dispersal of wild-type *H*. *pylori* PMSS1 following removal of HOCl point source. Micropipette contains 10 mM HOCl and 500 mM PBS (pH 6.7).
(MP4)

S13 Movie HOCl point-source assay with *tlpD H*. *pylori* G27. Micropipette contains 10 mM HOCl and 500 mM PBS (pH 6.7).
(MP4)

S14 Movie HOCl point-source assay with *tlpABC H*. *pylori* G27. Micropipette contains 10 mM HOCl and 500 mM PBS (pH 6.7).
(MP4)

S15 Movie HOCl point-source assay with *cheA H*. *pylori* G27. Micropipette contains 10 mM HOCl and 500 mM PBS (pH 6.7).
(MP4)

S1 Data Data values from main text and supplementary figures. (XLSX)

S1 Table Kinetics and dissociation constants of chemotaxis complex components. (DOCX)

S2 Table *H*. *pylori* swimming reversal rates in the presence of chemoeffectors. (DOCX)

S3 Table Key resources. (DOCX)

## Abbreviations

CD: circular dichroism
CheA: chemotaxis protein A
CheA-Pi: phosphorylated chemotaxis protein A
CheW: chemotaxis protein W
CheY: chemotaxis protein Y
CheY-Pi: phosphorylated chemotaxis protein Y
CZB: chemoreceptor zinc-binding; DgcZ, diguanylate cyclase Z
EDTA: Ethylenediaminetetraacetic acid
*Hp*TlpD: *Helicobacter pylori* transducer-like protein D
ICP: Inductively coupled plasma
KatA: catalase protein A
K_1/2_: the concentration at which 50% of the kinase was inactivated
Nrf2: nuclear factor erythroid 2–related factor 2
OHSU: Oregon Health and Science University
PBD: Protein Data Bank
RFU: relative fluorescence units
ROS: reactive oxygen species
TCEP: Tris(2-carboxyethyl)phosphine
Tlp: transducer-like protein
TlpD: transducer-like protein D
TPEN: N,N,N′,N′-tetrakis(2-pyridinylmethyl)-1,2-ethanediamine
YdeH: protein product of gene ydeH

## References

[1] Amieva M. and Peek R. M., “Pathobiology of Helicobacter pylori–Induced Gastric Cancer,” *Gastroenterology*, vol. 150, no. 1, pp. 64–78, 1 2016 10.1053/j.gastro.2015.09.004 26385073 PMC4691563

[2] Mégraud F., “The challenge of Helicobacter pylori resistance to antibiotics: the comeback of bismuth-based quadruple therapy,” *Therap Adv Gastroenterol*, vol. 5, no. 2, pp. 103–109, 3 2012 10.1177/1756283X11432492 22423259 PMC3296089

[3] Ramarao N., Gray‐Owen S. D., and Meyer T. F., “Helicobacter pylori induces but survives the extracellular release of oxygen radicals from professional phagocytes using its catalase activity,” *Molecular Microbiology*, vol. 38, no. 1, pp. 103–113, 10 2000 10.1046/j.1365-2958.2000.02114.x 11029693

[4] Kozol R. et al, “Neutrophil chemotaxis in gastric mucosa,” *Digest Dis Sci*, vol. 36, no. 9, pp. 1277–1280, 9 1991 10.1007/bf01307522 1893813

[5] Aihara E. et al., “Motility and Chemotaxis Mediate the Preferential Colonization of Gastric Injury Sites by Helicobacter pylori,” *PLoS Pathog*., vol. 10, no. 7, p. e1004275, 7 2014 10.1371/journal.ppat.1004275 25033386 PMC4102597

[6] Husson M. O., Legrand D., Spik G., and Leclerc H., “Iron acquisition by Helicobacter pylori: importance of human lactoferrin.,” *Infect Immun*, vol. 61, no. 6, pp. 2694–2697, 6 1993 8500909 10.1128/iai.61.6.2694-2697.1993 PMC280902

[7] Tan S., Noto J. M., Romero-Gallo J., Peek R. M., and Amieva M. R., “Helicobacter pylori perturbs iron trafficking in the epithelium to grow on the cell surface,” *PLoS Pathog*., vol. 7, no. 5, p. e1002050, 5 2011 10.1371/journal.ppat.1002050 21589900 PMC3093365

[8] Worst D. J., Otto B. R., and de Graaff J., “Iron-repressible outer membrane proteins of Helicobacter pylori involved in heme uptake.,” *Infection and Immunity*, vol. 63, no. 10, pp. 4161–4165, 10 1995 7558334 10.1128/iai.63.10.4161-4165.1995 PMC173585

[9] Huang J. Y., Sweeney E. G., Guillemin K., and Amieva M. R., “Multiple Acid Sensors Control Helicobacter pylori Colonization of the Stomach,” *PLoS Pathog*., vol. 13, no. 1, p. e1006118, 1 2017 10.1371/journal.ppat.1006118 28103315 PMC5245789

[10] Rolig A. S., Shanks J., Carter J. E., and Ottemann K. M., “Helicobacter pylori requires TlpD-driven chemotaxis to proliferate in the antrum,” *Infect*. *Immun*., p. IAI.00407–12, 7 2012. 10.1128/IAI.00407-12 PMC3457577 22802346

[11] “Colonization, localization, and inflammation: the roles of H. pylori chemotaxis in vivo,” *Current Opinion in Microbiology*, vol. 41, pp. 51–57, 2 2018 10.1016/j.mib.2017.11.019 29202336 PMC5862749

[12] Ortega Á., Zhulin I. B., and Krell T., “Sensory Repertoire of Bacterial Chemoreceptors,” *Microbiol*. *Mol*. *Biol*. *Rev*., vol. 81, no. 4, pp. e00033–17, 12 2017 10.1128/MMBR.00033-17 29070658 PMC5706747

[13] Hazelbauer G. L., Falke J. J., and Parkinson J. S., “Bacterial chemoreceptors: high-performance signaling in networked arrays,” *Trends in Biochemical Sciences*, vol. 33, no. 1, pp. 9–19, 1 2008 10.1016/j.tibs.2007.09.014 18165013 PMC2890293

[14] Briegel A., Li X., Bilwes A. M., Hughes K. T., Jensen G. J., and Crane B. R., “Bacterial chemoreceptor arrays are hexagonally packed trimers of receptor dimers networked by rings of kinase and coupling proteins,” *PNAS*, 2 2012. 10.1073/pnas.1115719109 PMC3309718 22355139

[15] Qin Z., Lin W., Zhu S., Franco A. T., and Liu J., “Imaging the Motility and Chemotaxis Machineries in Helicobacter pylori by Cryo-Electron Tomography,” *Journal of Bacteriology*, vol. 199, no. 3, pp. e00695–16, 2 2017 10.1128/JB.00695-16 27849173 PMC5237115

[16] Cassidy C. K. et al., “CryoEM and computer simulations reveal a novel kinase conformational switch in bacterial chemotaxis signaling,” *eLife*, 19-11-2015 [Online]. Available from: https://elifesciences.org/articles/08419. [cited 2018 Aug 21]. 10.7554/eLife.08419 PMC6746300 26583751

[17] Haglin E. R., Yang W., Briegel A., and Thompson L. K., “His-Tag-Mediated Dimerization of Chemoreceptors Leads to Assembly of Functional Nanoarrays,” *Biochemistry*, vol. 56, no. 44, pp. 5874–5885, 11 2017 10.1021/acs.biochem.7b00698 28872847 PMC5678893

[18] Zähringer F., Lacanna E., Jenal U., Schirmer T., and Boehm A., “Structure and Signaling Mechanism of a Zinc-Sensory Diguanylate Cyclase,” *Structure*, vol. 21, no. 7, pp. 1149–1157, 7 2013 10.1016/j.str.2013.04.026 23769666

[19] Crooks G. E., “WebLogo: A Sequence Logo Generator,” *Genome Research*, vol. 14, no. 6, pp. 1188–1190, 5 2004 10.1101/gr.849004 15173120 PMC419797

[20] Goers Sweeney E. et al, “Structure and Proposed Mechanism for the pH-Sensing Helicobacter pylori Chemoreceptor TlpB,” *Structure*, vol. 20, no. 7, pp. 1177–1188, 7 2012 10.1016/j.str.2012.04.021 22705207 PMC3392440

[21] Huang J. Y. et al., “Chemodetection and Destruction of Host Urea Allows Helicobacter pylori to Locate the Epithelium,” *Cell Host & Microbe*, vol. 18, no. 2, pp. 147–156, 8 2015. 26269952 10.1016/j.chom.2015.07.002 PMC4593702

[22] Machuca M. A., Johnson K. S., Liu Y. C., Steer D. L., Ottemann K. M., and Roujeinikova A., “Helicobacter pylori chemoreceptor TlpC mediates chemotaxis to lactate,” *Scientific Reports*, vol. 7, no. 1, p. 14089, 10 2017 10.1038/s41598-017-14372-2 29075010 PMC5658362

[23] Collins K. D., Lacal J., and Ottemann K. M., “Internal Sense of Direction: Sensing and Signaling from Cytoplasmic Chemoreceptors,” *Microbiol*. *Mol*. *Biol*. *Rev*., vol. 78, no. 4, pp. 672–684, 12 2014 10.1128/MMBR.00033-14 25428939 PMC4248653

[24] Briegel A. et al., “Structure of bacterial cytoplasmic chemoreceptor arrays and implications for chemotactic signaling,” *eLife*, vol. 3, 3 2014. 10.7554/eLife.02151 PMC3964821 24668172

[25] Draper J., Karplus K., and Ottemann K. M., “CZB: A Zinc-binding domain common to cytoplasmic bacterial chemoreceptors,” *J*. *Bacteriol*., p. JB.05140–11, 7 2011. 10.1128/JB.05140-11 PMC3165512 21725005

[26] Collins K. D. et al., “The Helicobacter pylori CZB cytoplasmic chemoreceptor TlpD forms an autonomous polar chemotaxis signaling complex that mediates a tactic response to oxidative stress,” *J*. *Bacteriol*., p. JB.00071–16, 3 2016. 10.1128/JB.00071-16 PMC4959281 27002127

[27] Behrens W. et al., “Localisation and protein-protein interactions of the Helicobacter pylori taxis sensor TlpD and their connection to metabolic functions,” *Scientific Reports*, vol. 6, p. 23582, 4 2016 10.1038/srep23582 27045738 PMC4820699

[28] Halliwell B., Clement M. V., and Long L. H., “Hydrogen peroxide in the human body,” *FEBS Letters*, vol. 486, no. 1, pp. 10–13, 12 2000 10.1016/s0014-5793(00)02197-9 11108833

[29] Schröder E. and Eaton P., “Hydrogen peroxide as an endogenous mediator and exogenous tool in cardiovascular research: issues and considerations,” *Current Opinion in Pharmacology*, vol. 8, no. 2, pp. 153–159, 4 2008 10.1016/j.coph.2007.12.012 18243791

[30] Schweinitzer T. et al., “Functional Characterization and Mutagenesis of the Proposed Behavioral Sensor TlpD of Helicobacter pylori,” *J*. *Bacteriol*., vol. 190, no. 9, pp. 3244–3255, 5 2008 10.1128/JB.01940-07 18245281 PMC2347378

[31] Altschul S. F., Gish W., Miller W., Myers E. W., and Lipman D. J., “Basic local alignment search tool,” *Journal of molecular biology*, vol. 215, no. 3, pp. 403–410, 1990 10.1016/S0022-2836(05)80360-2 2231712

[32] Zhang Y., “I-TASSER server for protein 3D structure prediction,” *BMC Bioinformatics*, vol. 9, p. 40, 1 2008 10.1186/1471-2105-9-40 18215316 PMC2245901

[33] Tawa P. and Stewart R. C., “Kinetics of CheA Autophosphorylation and Dephosphorylation Reactions,” *Biochemistry*, vol. 33, no. 25, pp. 7917–7924, 6 1994 10.1021/bi00191a019 8011654

[34] Gegner J. A., Graham D. R., Roth A. F., and Dahlquist F. W., “Assembly of an MCP receptor, CheW, and kinase CheA complex in the bacterial chemotaxis signal transduction pathway,” *Cell*, vol. 70, no. 6, pp. 975–982, 9 1992 10.1016/0092-8674(92)90247-a 1326408

[35] Gegner J. A. and Dahlquist F. W., “Signal transduction in bacteria: CheW forms a reversible complex with the protein kinase CheA,” *PNAS*, vol. 88, no. 3, pp. 750–754, 2 1991 10.1073/pnas.88.3.750 1992467 PMC50891

[36] Boukhvalova M. S., Dahlquist F. W., and Stewart R. C., “CheW Binding Interactions with CheA and Tar IMPORTANCE FOR CHEMOTAXIS SIGNALING IN ESCHERICHIA COLI,” *J*. *Biol*. *Chem*., vol. 277, no. 25, pp. 22251–22259, 6 2002 10.1074/jbc.M110908200 11923283

[37] Abedrabbo S., Castellon J., Collins K. D., Johnson K. S., and Ottemann K. M., “Cooperation of two distinct coupling proteins creates chemosensory network connections,” *PNAS*, p. 201618227, 2 2017. 10.1073/pnas.1618227114 PMC5358395 28242706

[38] Draper J., Karplus K., and Ottemann K. M., “Identification of a Chemoreceptor Zinc-Binding Domain Common to Cytoplasmic Bacterial Chemoreceptors,” *J*. *Bacteriol*., vol. 193, no. 17, pp. 4338–4345, 9 2011 10.1128/JB.05140-11 21725005 PMC3165512

[39] Clarke S. and Koshland D. E., “Membrane receptors for aspartate and serine in bacterial chemotaxis.,” *J*. *Biol*. *Chem*., vol. 254, no. 19, pp. 9695–9702, 10 1979 385590

[40] Stingl K., Uhlemann E.-M., Deckers-Hebestreit G., Schmid R., Bakker E. P., and Altendorf K., “Prolonged Survival and Cytoplasmic pH Homeostasis of Helicobacter pylori at pH 1,” *Infect Immun*, vol. 69, no. 2, pp. 1178–1180, 2 2001 10.1128/IAI.69.2.1178-1180.2001 11160017 PMC98001

[41] “Networked Chemoreceptors Benefit Bacterial Chemotaxis Performance | mBio.” [Online]. Available from: https://mbio.asm.org/content/7/6/e01824-16. [cited 2019 May 6]. 10.1128/mBio.01824-16 PMC5181776 27999161

[42] Mahawar M., Tran V., Sharp J. S., and Maier R. J., “Synergistic Roles of *Helicobacter pylori* Methionine Sulfoxide Reductase and GroEL in Repairing Oxidant-damaged Catalase,” *Journal of Biological Chemistry*, vol. 286, no. 21, pp. 19159–19169, 5 2011 10.1074/jbc.M111.223677 21460217 PMC3099729

[43] Poole L. B. and Nelson K. J., “Discovering mechanisms of signaling-mediated cysteine oxidation,” *Current Opinion in Chemical Biology*, vol. 12, no. 1, pp. 18–24, 2 2008 10.1016/j.cbpa.2008.01.021 18282483 PMC2408887

[44] Windle H. J., Fox A., Ní Eidhin D., and Kelleher D., “The thioredoxin system of Helicobacter pylori,” *J*. *Biol*. *Chem*., vol. 275, no. 7, pp. 5081–5089, 2 2000 10.1074/jbc.275.7.5081 10671551

[45] Seo Y. H. and Carroll K. S., “Profiling protein thiol oxidation in tumor cells using sulfenic acid-specific antibodies,” *Proceedings of the National Academy of Sciences*, vol. 106, no. 38, pp. 16163–16168, 9 2009. 10.1073/pnas.0903015106 PMC2741475 19805274

[46] Perkins A., Nelson K. J., Williams J. R., Parsonage D., Poole L. B., and Karplus P. A., “The Sensitive Balance Between the Fully Folded and Locally Unfolded Conformations of a Model Peroxiredoxin,” *Biochemistry*, vol. 52, no. 48, pp. 8708–8721, 12 2013 10.1021/bi4011573 24175952 PMC3932808

[47] Perkins A., Nelson K. J., Parsonage D., Poole L. B., and Karplus P. A., “Peroxiredoxins: guardians against oxidative stress and modulators of peroxide signaling,” *Trends in Biochemical Sciences*, vol. 40, no. 8, pp. 435–445, 8 2015 10.1016/j.tibs.2015.05.001 26067716 PMC4509974

[48] Perkins A., Poole L. B., and Karplus P. A., “Tuning of peroxiredoxin catalysis for various physiological roles,” *Biochemistry*, vol. 53, no. 49, pp. 7693–7705, 12 2014 10.1021/bi5013222 25403613 PMC4270387

[49] Weiss S. J., “Tissue destruction by neutrophils,” *New England Journal of Medicine*, vol. 320, no. 6, pp. 365–376, 1989 10.1056/NEJM198902093200606 2536474

[50] Young I., “Proof without prejudice: use of the Kolmogorov-Smirnov test for the analysis of histograms from flow systems and other sources.,” *Journal of Histochemistry & Cytochemistry*, vol. 25, no. 7, pp. 935–941, 7 1977. 894009 10.1177/25.7.894009

[51] Parthasarathy R., “Rapid, accurate particle tracking by calculation of radial symmetry centers,” *Nature Methods*, vol. 9, no. 7, pp. 724–726, 7 2012 10.1038/nmeth.2071 22688415

[52] Howitt M. R. et al., “ChePep controls Helicobacter pylori Infection of the gastric glands and chemotaxis in the Epsilonproteobacteria,” *MBio*, vol. 2, no. 4, 2011. 10.1128/mBio.00098-11 PMC3143842 21791582

[53] Lebrun V., Ravanat J.-L., Latour J.-M., and Sénèque O., “Near diffusion-controlled reaction of a Zn(Cys) 4 zinc finger with hypochlorous acid,” *Chemical Science*, vol. 7, no. 8, pp. 5508–5516, 2016 10.1039/c6sc00974c 30034691 PMC6021785

[54] Bourlès E., Isaac M., Lebrun C., Latour J.-M., and Sénèque O., “Oxidation of Zn(Cys)4 zinc finger peptides by O2 and H2O2: products, mechanism and kinetics,” *Chemistry*, vol. 17, no. 49, pp. 13762–13772, 12 2011 10.1002/chem.201101913 22052717

[55] Crow J. P., Beckman J. S., and McCord J. M., “Sensitivity of the Essential Zinc-Thiolate Moiety of Yeast Alcohol Dehydrogenase to Hypochlorite and Peroxynitrite,” *Biochemistry*, vol. 34, no. 11, pp. 3544–3552, 3 1995 10.1021/bi00011a008 7893650

[56] Springman E. B., Angleton E. L., Birkedal-Hansen H., and Wart H. E. V., “Multiple modes of activation of latent human fibroblast collagenase: evidence for the role of a Cys73 active-site zinc complex in latency and a ‘cysteine switch’ mechanism for activation,” *PNAS*, vol. 87, no. 1, pp. 364–368, 1 1990 10.1073/pnas.87.1.364 2153297 PMC53264

[57] Van Wart H. E. and Birkedal-Hansen H., “The cysteine switch: a principle of regulation of metalloproteinase activity with potential applicability to the entire matrix metalloproteinase gene family.,” *Proceedings of the National Academy of Sciences*, vol. 87, no. 14, pp. 5578–5582, 7 1990. 10.1073/pnas.87.14.5578 PMC54368 2164689

[58] Pullar J., Vissers M., and Winterbourn C., “Living with a Killer: The Effects of Hypochlorous Acid on Mammalian Cells,” *IUBMB Life*, vol. 50, no. 4, pp. 259–266, 10 2001. 10.1080/713803731 11327319

[59] Gray M. J., Wholey W.-Y., and Jakob U., “Bacterial Responses to Reactive Chlorine Species,” *Annu Rev Microbiol*, vol. 67, pp. 141–160, 2013 10.1146/annurev-micro-102912-142520 23768204 PMC3891400

[60] Ganesana M., Erlichman J. S., and Andreescu S., “Real-time monitoring of superoxide accumulation and antioxidant activity in a brain slice model using an electrochemical cytochrome c biosensor,” *Free Radic Biol Med*, vol. 53, no. 12, 12 2012. 10.1016/j.freeradbiomed.2012.10.540 PMC3565046 23085519

[61] Test S. T. and Weiss S. J., “The Generation of Utilization of Chlorinated Oxidants by Human NeutrophUs,” p. 26.

[62] Tatsumi T. and Fliss H., “Hypochlorous acid and chloramines increase endothelial permeability: possible involvement of cellular zinc,” *American Journal of Physiology-Heart and Circulatory Physiology*, vol. 267, no. 4, pp. H1597–H1607, 10 1994. 10.1152/ajpheart.1994.267.4.H1597 7943407

[63] Gray M. J., Wholey W.-Y., Parker B. W., Kim M., and Jakob U., “NemR Is a Bleach-sensing Transcription Factor,” *J*. *Biol*. *Chem*., vol. 288, no. 19, pp. 13789–13798, 5 2013 10.1074/jbc.M113.454421 23536188 PMC3650415

[64] Stent A., Every A. L., and Sutton P., “Helicobacter pylori defense against oxidative attack,” *American Journal of Physiology-Gastrointestinal and Liver Physiology*, vol. 302, no. 6, pp. G579–G587, 12 2011 10.1152/ajpgi.00495.2011 22194421

[65] Dixon M. F., Genta R. M., Yardley J. H., and Correa P., “Classification and grading of gastritis. The updated Sydney System. International Workshop on the Histopathology of Gastritis, Houston 1994,” *Am*. *J*. *Surg*. *Pathol*., vol. 20, no. 10, pp. 1161–1181, 10 1996 8827022 10.1097/00000478-199610000-00001

[66] Lee I., “Critical pathogenic steps to high risk Helicobacter pylori gastritis and gastric carcinogenesis,” *World J*. *Gastroenterol*., vol. 20, no. 21, pp. 6412–6419, 6 2014 10.3748/wjg.v20.i21.6412 24914362 PMC4047326

[67] Sigal M. et al., “Helicobacter pylori Activates and Expands Lgr5(+) Stem Cells Through Direct Colonization of the Gastric Glands,” *Gastroenterology*, vol. 148, no. 7, p. 1392–1404.e21, 6 2015 10.1053/j.gastro.2015.02.049 25725293

[68] Fung C. et al., “High-resolution mapping reveals that microniches in the gastric glands control Helicobacter pylori colonization of the stomach,” *PLoS Biol*., vol. 17, no. 5, p. e3000231, 5 2019 10.1371/journal.pbio.3000231 31048876 PMC6497225

[69] Keilberg D., Zavros Y., Shepherd B., Salama N. R., and Ottemann K. M., “Spatial and Temporal Shifts in Bacterial Biogeography and Gland Occupation during the Development of a Chronic Infection,” *MBio*, vol. 7, no. 5, 11 2016. 10.1128/mBio.01705-16 PMC5061875 27729513

[70] Suzuki H., Nishizawa T., Tsugawa H., Mogami S., and Hibi T., “Roles of oxidative stress in stomach disorders,” *J Clin Biochem Nutr*, vol. 50, no. 1, pp. 35–39, 1 2012 10.3164/jcbn.11-115SR 22247598 PMC3246180

[71] Thao T. D. H., Ryu H.-C., Yoo S.-H., and Rhee D.-K., “Antibacterial and anti-atrophic effects of a highly soluble, acid stable UDCA formula in Helicobacter pylori-induced gastritis,” *Biochemical Pharmacology*, vol. 75, no. 11, pp. 2135–2146, 6 2008 10.1016/j.bcp.2008.03.008 18436193

[72] Yanaka A. et al., “Dietary sulforaphane-rich broccoli sprouts reduce colonization and attenuate gastritis in Helicobacter pylori-infected mice and humans,” *Cancer Prev Res (Phila)*, vol. 2, no. 4, pp. 353–360, 4 2009. 19349290 10.1158/1940-6207.CAPR-08-0192

[73] Litvak Y., Byndloss M. X., Tsolis R. M., and Bäumler A. J., “Dysbiotic Proteobacteria expansion: a microbial signature of epithelial dysfunction,” *Curr*. *Opin*. *Microbiol*., vol. 39, pp. 1–6, 10 2017 10.1016/j.mib.2017.07.003 28783509

[74] Paiva C. N. and Bozza M. T., “Are Reactive Oxygen Species Always Detrimental to Pathogens?,” *Antioxid Redox Signal*, vol. 20, no. 6, pp. 1000–1037, 2 2014 10.1089/ars.2013.5447 23992156 PMC3924804

[75] Bäumler A. J. and Sperandio V., “Interactions between the microbiota and pathogenic bacteria in the gut,” *Nature*, vol. 535, no. 7610, pp. 85–93, 7 2016 10.1038/nature18849 27383983 PMC5114849

[76] Diaz-Ochoa V. E. et al., “Salmonella mitigates oxidative stress and thrives in the inflamed gut by evading calprotectin-mediated manganese sequestration,” *Cell Host Microbe*, vol. 19, no. 6, pp. 814–825, 6 2016 10.1016/j.chom.2016.05.005 27281571 PMC4901528

[77] Stecher B. et al., “Salmonella enterica serovar typhimurium exploits inflammation to compete with the intestinal microbiota,” *PLoS Biol*., vol. 5, no. 10, pp. 2177–2189, 10 2007 10.1371/journal.pbio.0050244 17760501 PMC1951780

[78] Thiennimitr P. et al., “Intestinal inflammation allows Salmonella to use ethanolamine to compete with the microbiota,” *Proc*. *Natl*. *Acad*. *Sci*. *U*.*S*.*A*., vol. 108, no. 42, pp. 17480–17485, 10 2011 10.1073/pnas.1107857108 21969563 PMC3198331

[79] Atsriku C., Scott G. K., Benz C. C., and Baldwin M. A., “Reactivity of Zinc Finger Cysteines: Chemical Modifications Within Labile Zinc Fingers in Estrogen Receptor,” *Journal of the American Society for Mass Spectrometry*, vol. 16, no. 12, pp. 2017–2026, 12 2005 10.1016/j.jasms.2005.08.009 16246571

[80] Hao Q. and Maret W., “Aldehydes release zinc from proteins. A pathway from oxidative stress/lipid peroxidation to cellular functions of zinc,” *The FEBS Journal*, vol. 273, no. 18, pp. 4300–4310, 2006 10.1111/j.1742-4658.2006.05428.x 16930132

[81] Behrens W. et al., “Role of Energy Sensor TlpD of Helicobacter pylori in Gerbil Colonization and Genome Analyses after Adaptation in the Gerbil,” *Infection and Immunity*, vol. 81, no. 10, pp. 3534–3551, 10 2013 10.1128/IAI.00750-13 23836820 PMC3811781

[82] Collins K. D., Hu S., Grasberger H., Kao J. Y., and Ottemann K. M., “Chemotaxis allows bacteria to overcome host-generated reactive oxygen species that constrain gland colonization,” *Infect*. *Immun*., p. IAI.00878–17, 3 2018. 10.1128/IAI.00878-17 PMC5913845 29507083

[83] Collins K. D., Hu S., Grasberger H., Kao J. Y., and Ottemann K. M., “Chemotaxis allows bacteria to overcome host-generated reactive oxygen species that constrain gland colonization,” *Infect*. *Immun*., p. IAI.00878–17, 3 2018. 10.1128/IAI.00878-17 PMC5913845 29507083

[84] “Disruption of the Epithelial Apical-Junctional Complex by Helicobacter pylori CagA | Science.” [Online]. Available from: https://science.sciencemag.org/content/300/5624/1430?casa_token=ZgcMEWKTxXAAAAAA:sBcs6-hvQEFYyFjHuYZeh783__56WRB4h8pfUVPB4-kaCk1u1Z3WYxcSV44seuSOwrDmeZD69_Pffg. [cited 2019 May 10]. 10.1126/science.1081919 PMC3369828 12775840

[85] “Host-Bacterial Interactions in Helicobacter pylori Infection—ScienceDirect.” [Online]. Available from: https://www.sciencedirect.com/science/article/abs/pii/S0016508507020161. [cited 2019 May 10].

[86] “Helicobacter pylori Usurps Cell Polarity to Turn the Cell Surface into a Replicative Niche.” [Online]. Available from: https://journals.plos.org/plospathogens/article?id=10.1371/journal.ppat.1000407. [cited 2019 May 10]. 10.1371/journal.ppat.1000407 PMC2669173 19412339

[87] “Helicobacter pylori VacA, a paradigm for toxin multifunctionality | Nature Reviews Microbiology.” [Online]. Available from: https://www.nature.com/articles/nrmicro1095. [cited 2019 May 10]. 10.1038/nrmicro1095 15759043

[88] Anderson J. K. et al., “Chemorepulsion from the Quorum Signal Autoinducer-2 Promotes Helicobacter pylori Biofilm Dispersal,” *mBio*, vol. 6, no. 4, pp. e00379–15, 9 2015 10.1128/mBio.00379-15 26152582 PMC4488943

[89] Liu X. and Parales R. E., “Chemotaxis of Escherichia coli to Pyrimidines: a New Role for the Signal Transducer Tap,” *Journal of Bacteriology*, vol. 190, no. 3, pp. 972–979, 2 2008 10.1128/JB.01590-07 18065551 PMC2223585

[90] Sievers F. et al., “Fast, scalable generation of high‐quality protein multiple sequence alignments using Clustal Omega,” *Molecular Systems Biology*, vol. 7, no. 1, p. 539, 1 2011. 21988835 10.1038/msb.2011.75 PMC3261699

[91] Guindon S., Dufayard J.-F., Lefort V., Anisimova M., Hordijk W., and Gascuel O., “New Algorithms and Methods to Estimate Maximum-Likelihood Phylogenies: Assessing the Performance of PhyML 3.0,” *Systematic Biology*, vol. 59, no. 3, pp. 307–321, 3 2010 10.1093/sysbio/syq010 20525638

[92] Furman C. S. and Margerum D. W., “Mechanism of Chlorine Dioxide and Chlorate Ion Formation from the Reaction of Hypobromous Acid and Chlorite Ion,” *Inorg Chem*, vol. 37, no. 17, pp. 4321–4327, 8 1998 11670568 10.1021/ic980262q

[93] Hess J. F., Oosawa K., Kaplan N., and Simon M. I., “Phosphorylation of three proteins in the signaling pathway of bacterial chemotaxis,” *Cell*, vol. 53, no. 1, pp. 79–87, 4 1988 10.1016/0092-8674(88)90489-8 3280143

[94] Lebowitz J., Lewis M. S., and Schuck P., “Modern analytical ultracentrifugation in protein science: A tutorial review,” *Protein Science*, vol. 11, no. 9, pp. 2067–2079, 9 2002 10.1110/ps.0207702 12192063 PMC2373601

[95] Collins T. J., “ImageJ for microscopy,” *BioTechniques*, vol. 43, no. 1S, pp. S25–S30, 7 2007. 10.2144/000112517 17936939

