# Supplementary figures and images for: Helicobacter pylori senses bleach (HOCl) as a chemoattractant using a cytosolic chemoreceptor
Source: PLoS Biol. 2019 Aug 29;17(8):e3000395. doi: 10.1371/journal.pbio.3000395 (PMC6715182; doi:10.1371/journal.pbio.3000395)

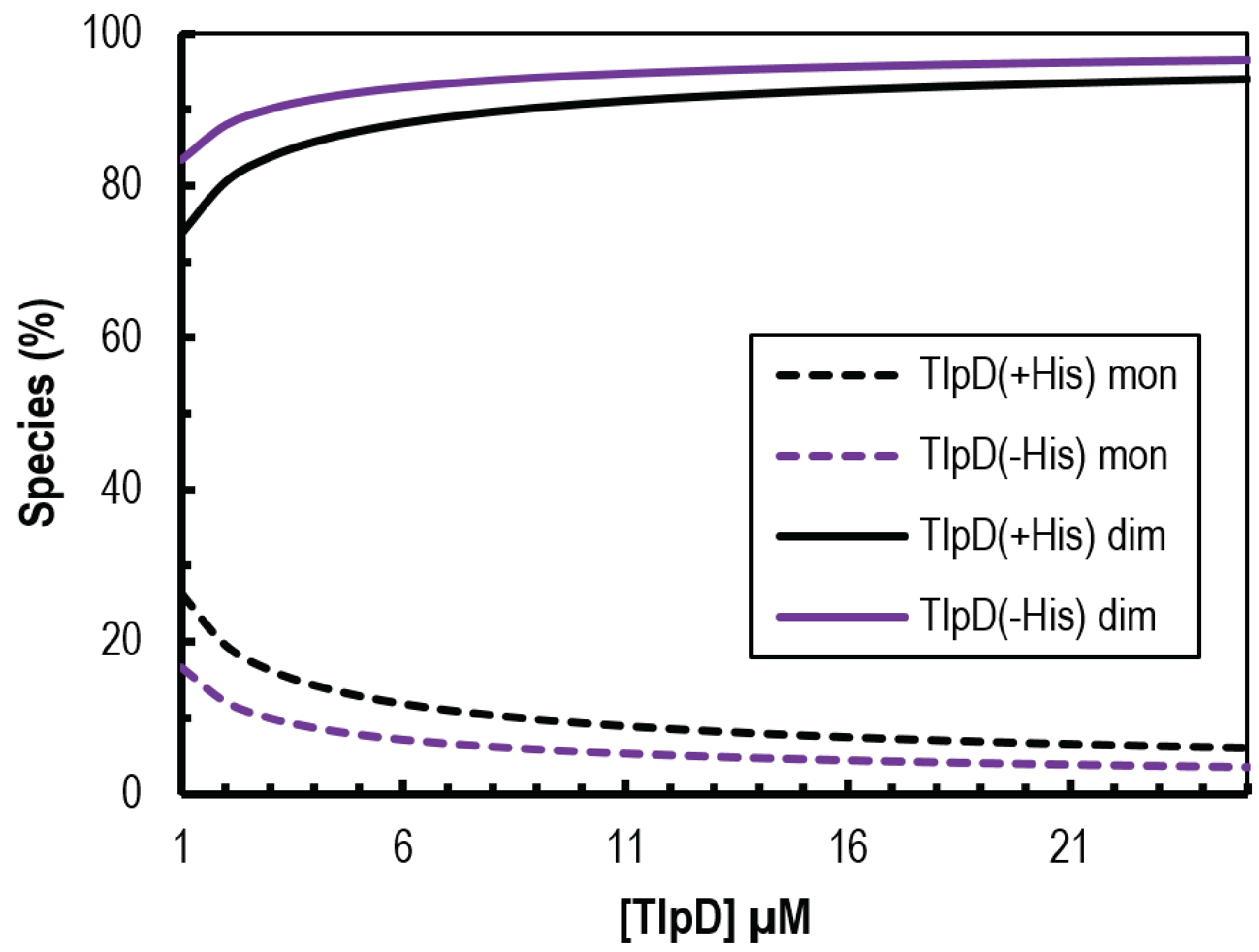

Supplement: S1 Fig — Formation of TlpD homodimers in a buffer of PBS (pH 7) and 1 mM TCEP was analyzed in a series of AUC experiments for recombinant TlpD with either an intact N-terminal His tag, TlpD(+His), or with the tag cleaved by TEV protease, TlpD(-His). The TlpD(+His) and TlpD(-His) KD values were found to be 188.5 nM SEM ± 121.4 nM and 64.8 nM SEM ± 2.1 nM, respectively. Relative amounts of monomer (dashed lines) and dimer (solid lines) estimated from these values are shown for TlpD(+His) (black) and TlpD(-His) forms as a function of total protein concentration. TCEP, Tris(2-carboxyethyl)phosphine; TlpD, transducer-like protein D. (TIF) [file pbio.3000395.s001.tif]

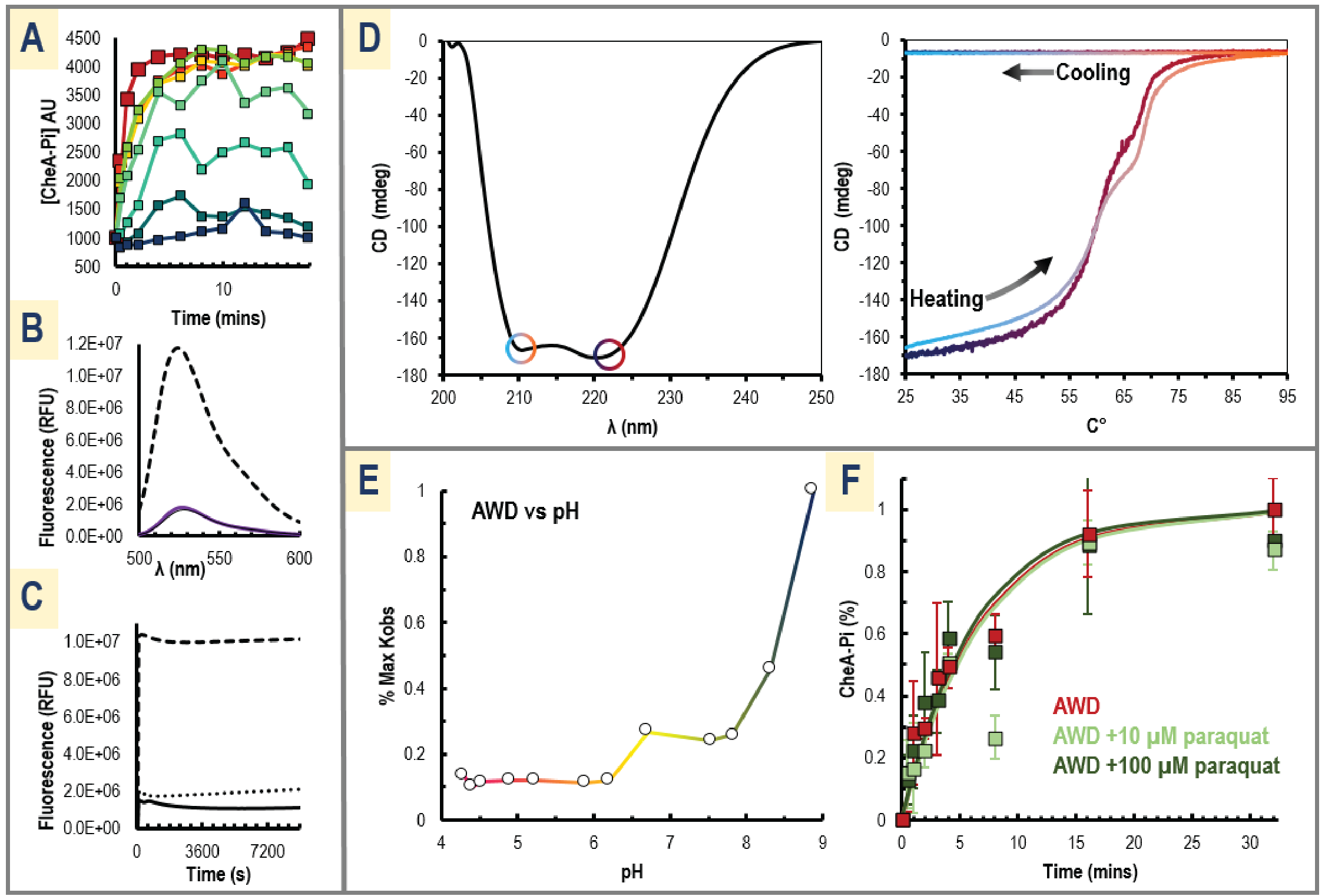

Supplement: S2 Fig — (A) A series of functional assays using reconstituted TlpD-CheW-CheA signaling complex with addition of 0 (red), 0.25× (orange), 0.5× (yellow), 1× (lime green), 2× (teal), 4× (cyan), 8× (dark green), and 16× (dark blue) zinc sulfate relative to [TlpD]. Decreases in activity were due to protein precipitation. (B) Shown is a fluorescence emission spectrum (ex. 492 nm) using 50 μM Zinpyr-1 in which the addition of 300 μM zinc acetate exhibits an increase in fluorescence at 547 nm (black dashes), but the addition of 30 μM TlpD for 10 minutess (purple) does not increase fluorescence over Zinpyr-1 alone (black, closely overlays with purple). (C) Time courses monitoring fluorescence (ex. 429/em. 527 nm) for 50 μM of the Zn-chelating probe Zinpyr-1 with a positive control of 50 μM zinc acetate (black dashes),versus 50 μM TlpD (black solid) or 50 μM heat-denatured TlpD (black dotted). (D) Shown left is a CD spectra of 50 μM TlpD in a buffer of 150 mM NaCl and 17.5 mM sodium citrate (pH 7). Shown right is a melting curve in which the sample was heated from 25°C to 95°C and then cooled back to 25°C with the CD at 210 nm (blue-orange) and 225 nm (blue-red) monitored as a function of temperature. (E) Shown are results from a series of functional assays showing relative rates of CheA autophosphorylation at low (pink), neutral (yellow), and basic (blue) pH. Experiments were run with 1 mM ATP, 4 μM CheA, 8 μM CheW, and 24 μM TlpD with 10 mM MgCl2, 100 mM NaCl, and 200 mM of total buffer comprised of a combination of sodium citrate and tris. (F) Functional assays are shown with either 1-hour pretreatment with buffer (red), 10 μM paraquat (light green), or 100 μM paraquat (dark green). CD, circular dichroism; CheA, chemotaxis protein A; CheW, chemotaxis protein W; em., emission; ex., excitation; TlpD, transducer-like protein D. (TIF) [file pbio.3000395.s002.tif]

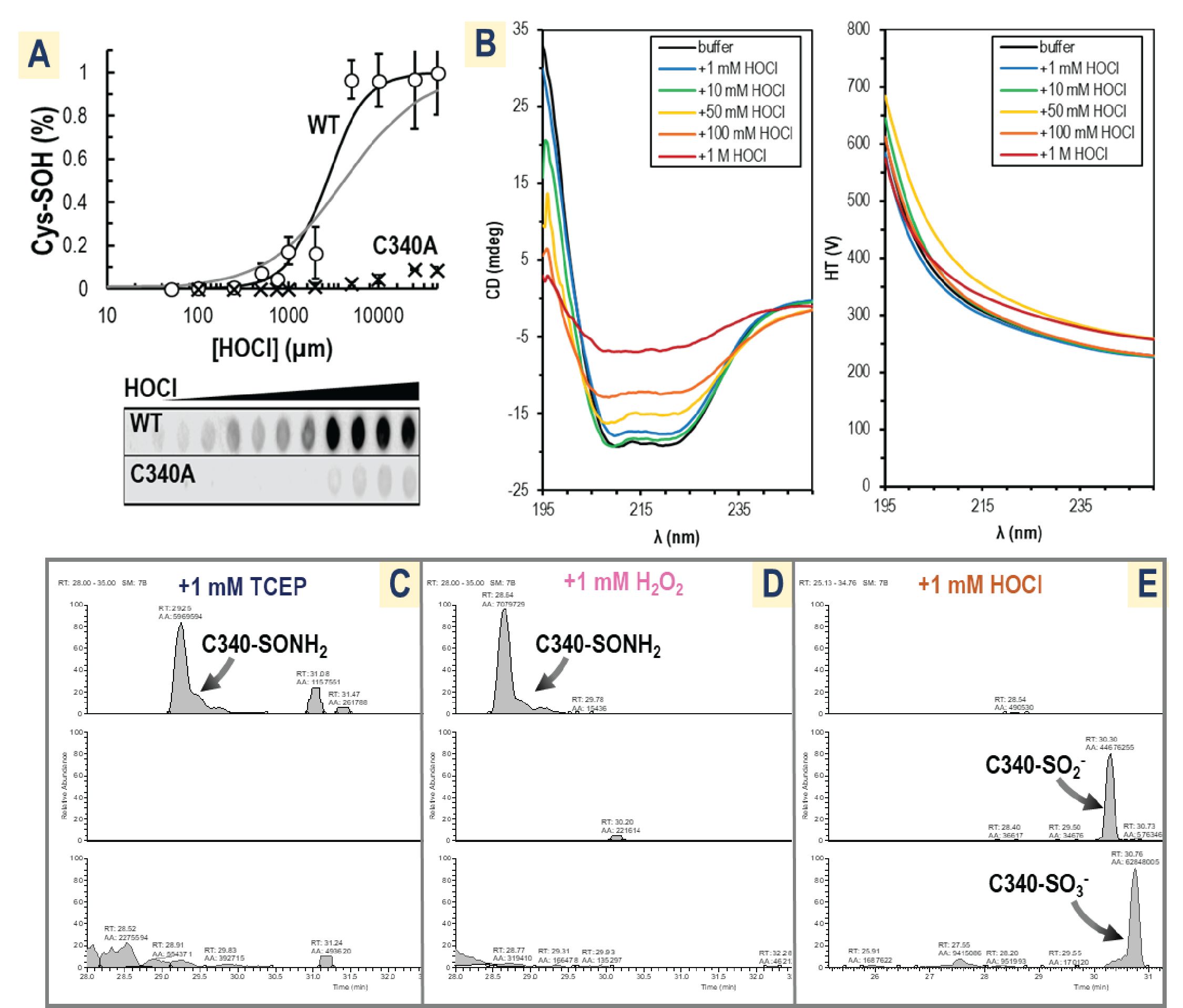

Supplement: S3 Fig — (A) Data from Fig 4D shown with a logarithmic x-axis. (B) Shown is a titration of HOCl against 5 μM TlpD monitored by CD. Samples were treated at the concentrations indicated for 10 minutes in a buffer of PBS (pH 7) and then desalted into a buffer of 150 mM NaCl and 17.5 mM sodium citrate (pH 7) prior to analysis to reduce voltage and facilitate CD measurement. (C–E) Shown are MS/MS ion extractions for the TlpD C340-containing peptide NCRLGKWYYEGAGK from samples treated with 1 mM TCEP (C), hydrogen peroxide (D), or HOCl (E). For each sample, ion extractions for +3 charged peptides containing modifications corresponding to alkylation are shown on top (C340-SONH2, unreacted cysteine thiols modified by iodoacetamide), oxidation of C340 to a cysteine sulfinate (C340-SO2−) are shown in the middle, and oxidation of C340 to a cysteine sulfonate (C340-SO3−) are shown on the bottom on a fixed scale. Integrations values for peaks are noted with “AA.” The experiment and analysis are described further in the Method details. CD, circular dichroism; MS/MS, tandem mass spectrometry; TCEP, Tris(2-carboxyethyl)phosphine; TlpD, transducer-like protein D. (TIF) [file pbio.3000395.s003.tif]

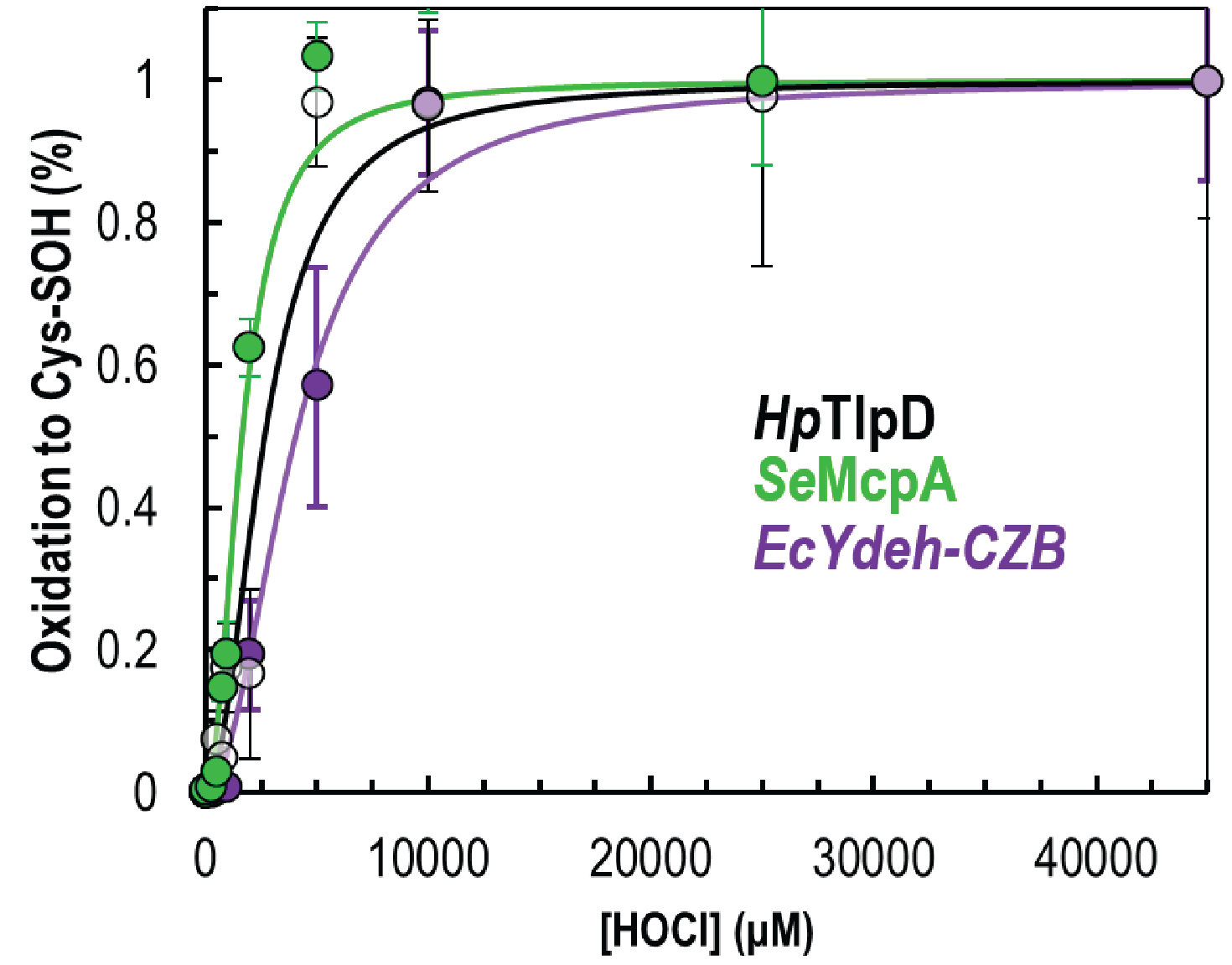

Supplement: S4 Fig — CZB protein domains from different bacterial species were analyzed for reactivity toward HOCl and formation of cysteine sulfenic acid. Shown are reactions of purified HpTlpD (data from Fig 4D, black line and open circles), S. enterica McpA (green), and the CZB domain of E. coli DgcZ (also called YdeH) with various concentrations of HOCl, run as in Fig 4D. Solid lines are fits of the data to the Hill equation with a coefficient of 2, and markers shown are the average of triplicate independent measurements. Error bars are the sample standard deviation. CZB, chemoreceptor zinc-binding; DgcZ, diguanylate cyclase Z; HpTlpD, Helicobacter pylori transducer-like protein D; YdeH,protein product of gene ydeH. (TIF) [file pbio.3000395.s004.tif]

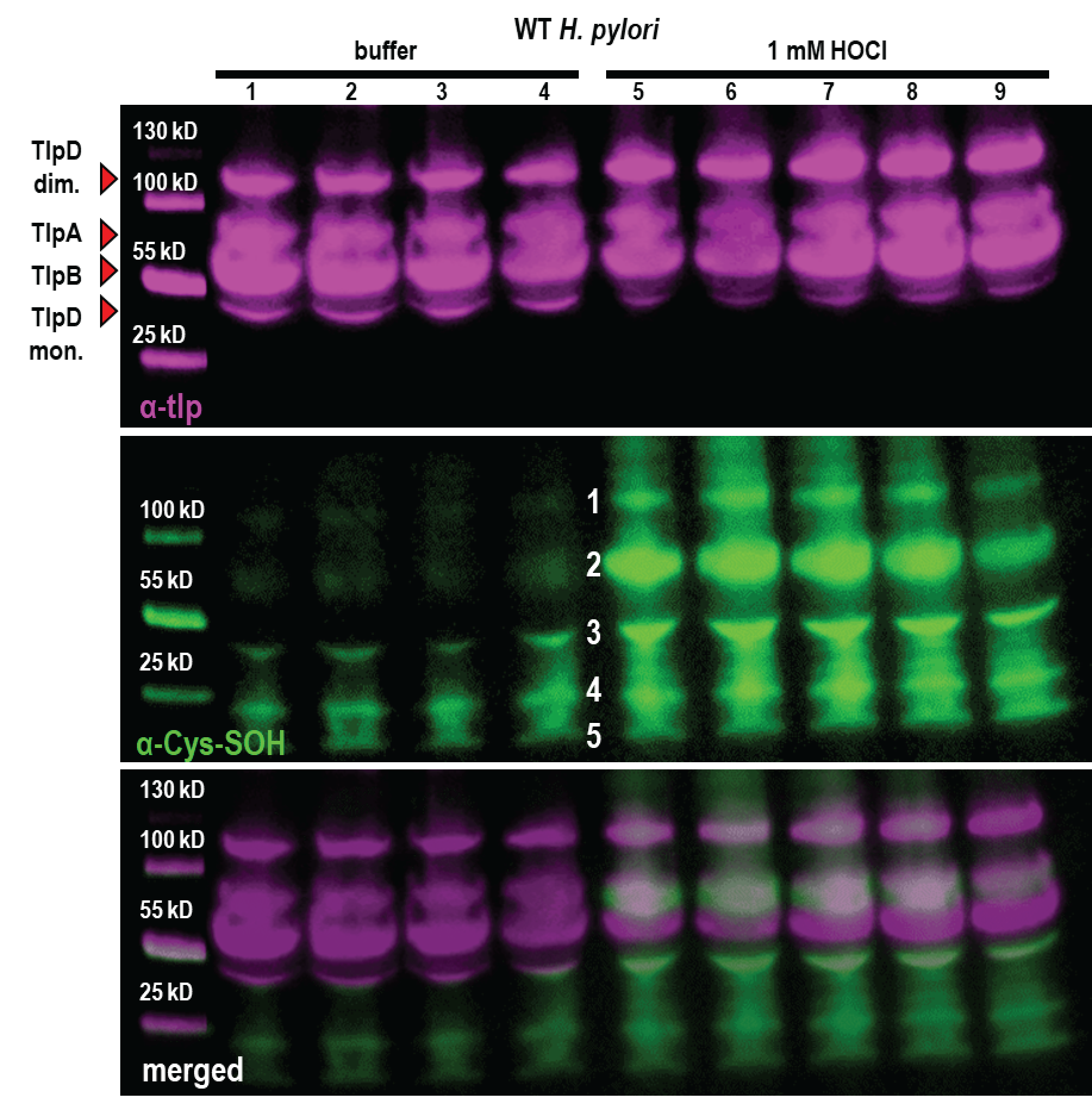

Supplement: S5 Fig — Shown is a single western blot of independent treatments of H. pylori G27 cells with PBS buffer (pH 7; lanes 1–4) or 1 mM HOCl in PBS buffer (pH 7; lanes 5–9). The blot was probed with α-Tlp primary antibody (pink), stripped, and reprobed with α-Cys-SOH (green). The resulting chemiluminescence images from each probe are shown overlaid and merged on the bottom. The protein ladder for each image was collected on a separate channel and overlaid with the chemiluminescence data. See Fig 5B and Method details for additional information. WT, wild type. (TIF) [file pbio.3000395.s005.tif]

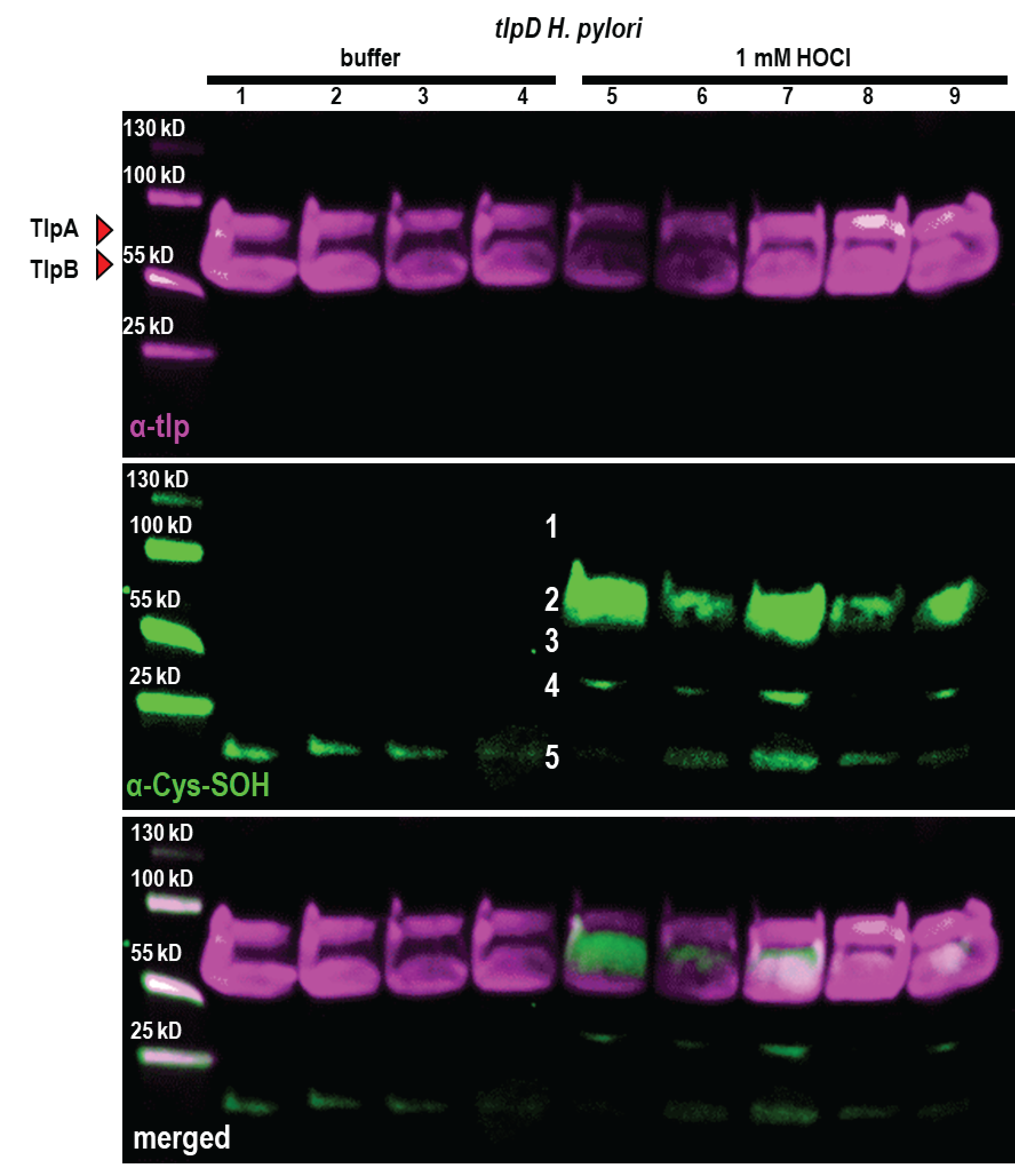

Supplement: S6 Fig — Shown as in S5A Fig, single western blot of independent treatments of H. pylori tlpD G27 cells with PBS buffer (pH 7; lanes 1–4) or 1 mM HOCl in PBS buffer (pH 7; lanes 5–9). (TIF) [file pbio.3000395.s006.tif]

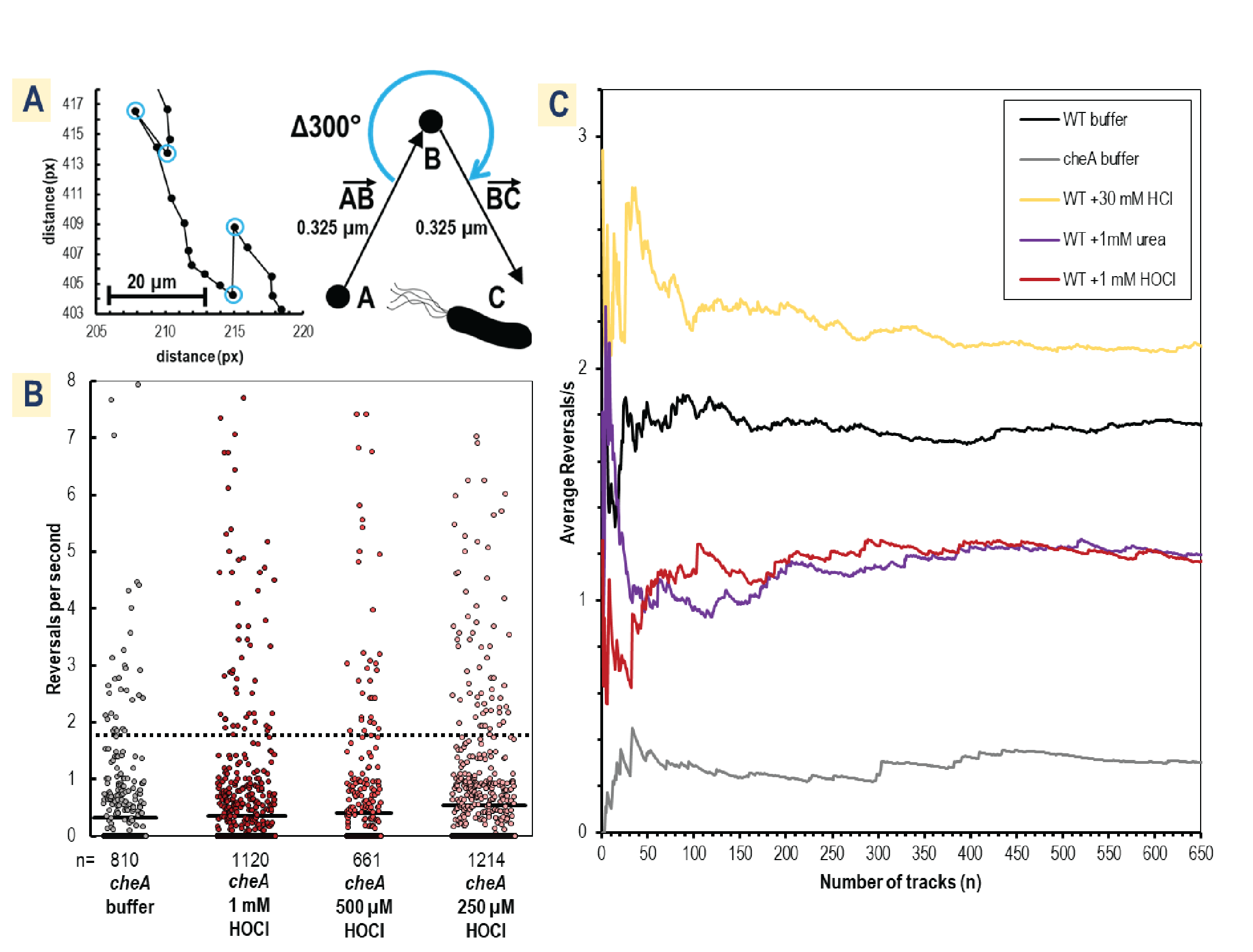

Supplement: S7 Fig — (A) Shown left is a representative swimming track for wild-type H. pylori G27 with each dot corresponding to the bacteria’s position every 40 ms. Reversals are highlighted as blue circles. Shown right is the definition of a reversal applied for quantification in this study and in Fig 5. (B) Control experiments with cheA H. pylori are shown with identical buffer and HOCl treatments as in Fig 5. Data shown are from 3 independent experiments. The baseline of the wild-type reversal rate treated with PBS buffer (pH 7) is indicated as a black dashed line. Black solid lines indicate the mean reversal rate under each condition. Because cheA is a chemotaxis-null mutant, these values can be considered as the false-positive rate of a fully smooth-swimming population, in which the swimming trajectory by chance elicits a trajectory change indistinguishable from a chemotactic reversal. (C) Shown is the mean reversal rate for representative data sets versus number of bacteria tracks. (TIF) [file pbio.3000395.s007.tif]

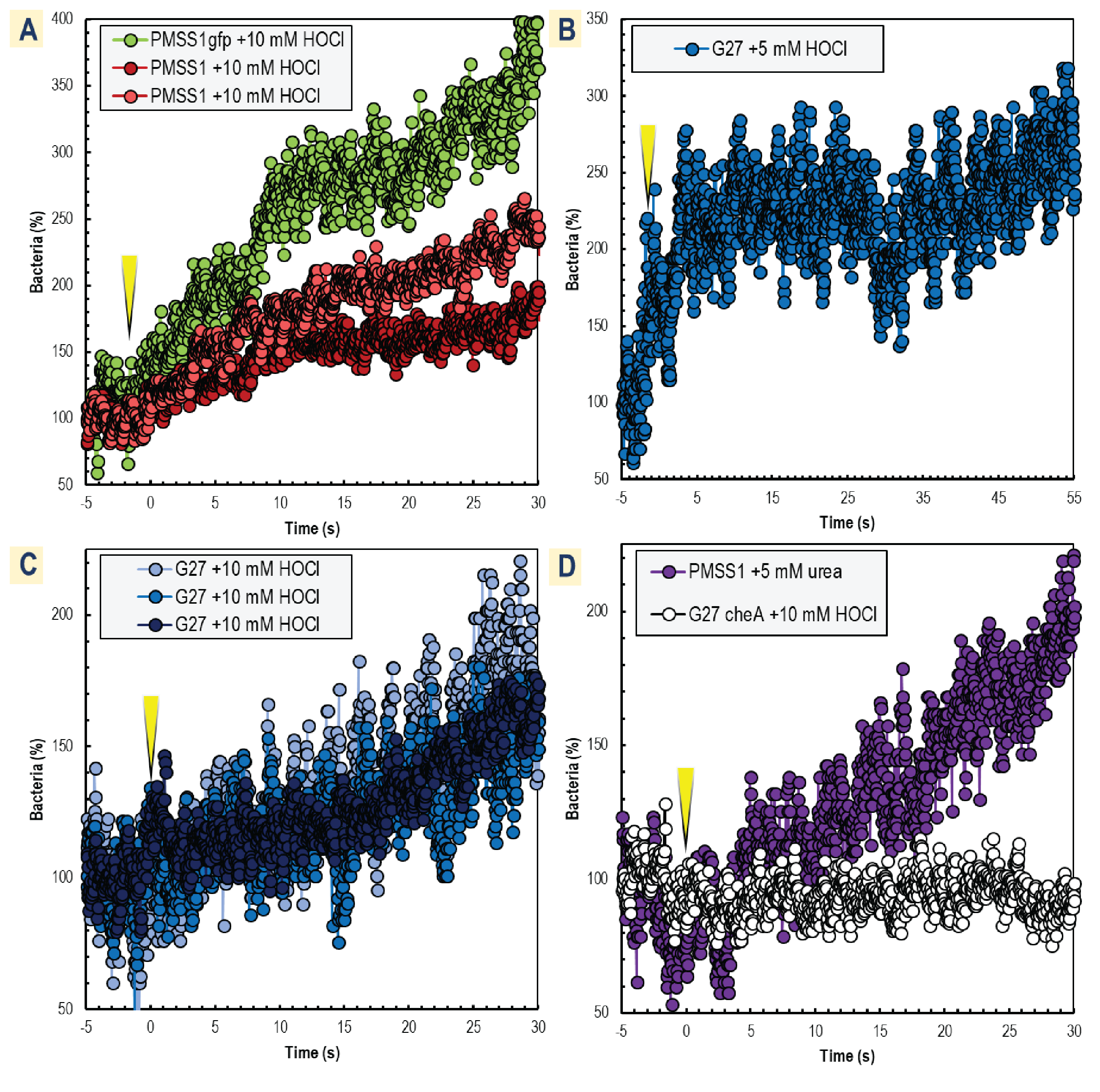

Supplement: S8 Fig — (A–C) Shown are data from representative chemotaxis experiments collected on different days showing the responses of G27 and PMSS1 H. pylori to a micropipette point source containing a solution of 5 to 10 mM HOCl and 500 mM PBS (pH 6.7). Data shown are relative bacteria counts in the frame of view during pre- and post-treatment for each frame of video (30 fps) normalized to counts prior to treatment. Treatment begins at time 0, indicated with a yellow arrow. (D) Additional controls showing response to the chemoattractant urea and the lack of a response to HOCl for a chemotaxis-null cheA mutant. (TIF) [file pbio.3000395.s008.tif]

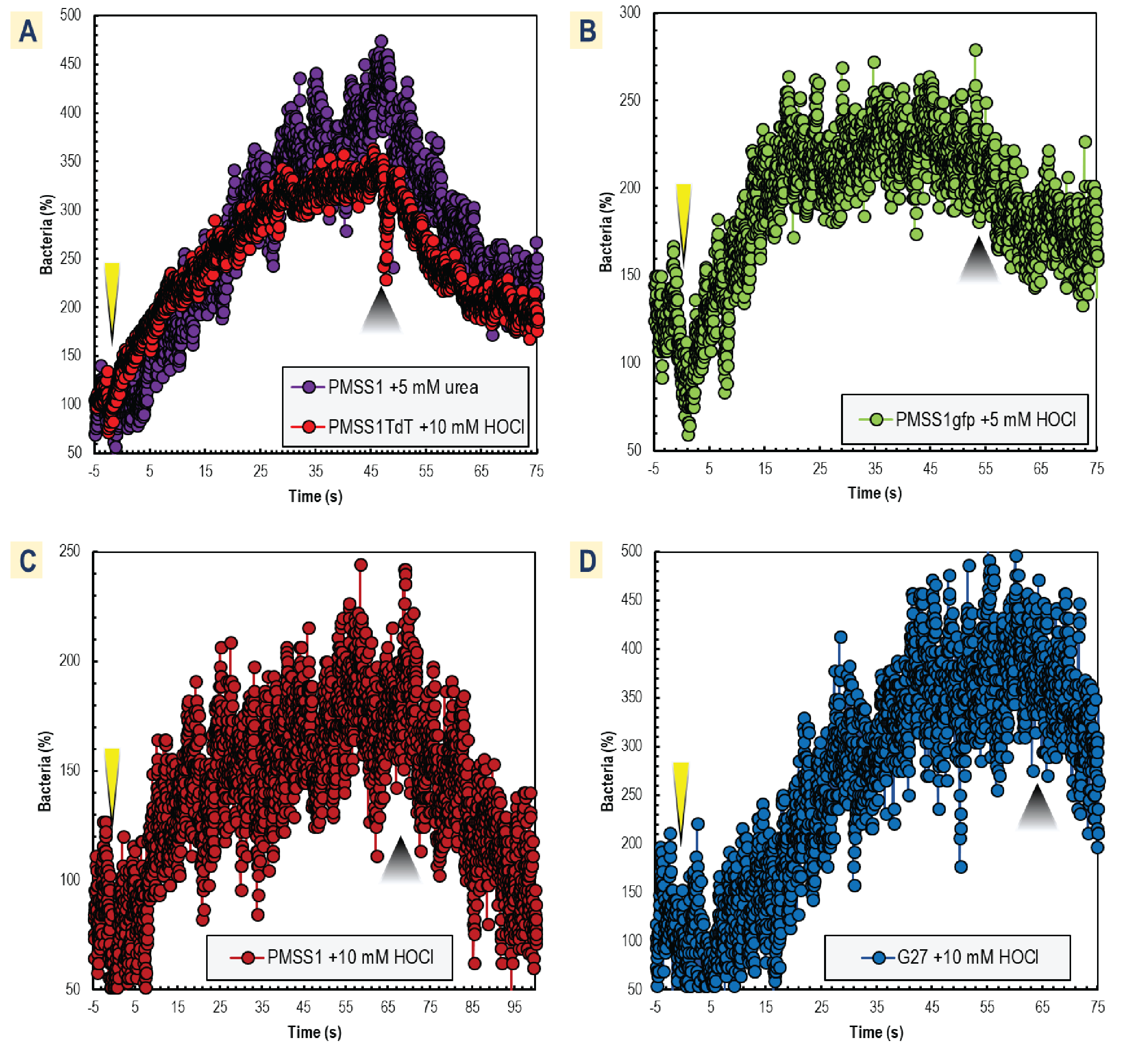

Supplement: S9 Fig — (A–D) Shown are data from representative point-source chemotaxis assays in which the point source is removed after the accumulation of bacteria in the field of view (indicated by black arrows). (TIF) [file pbio.3000395.s009.tif]

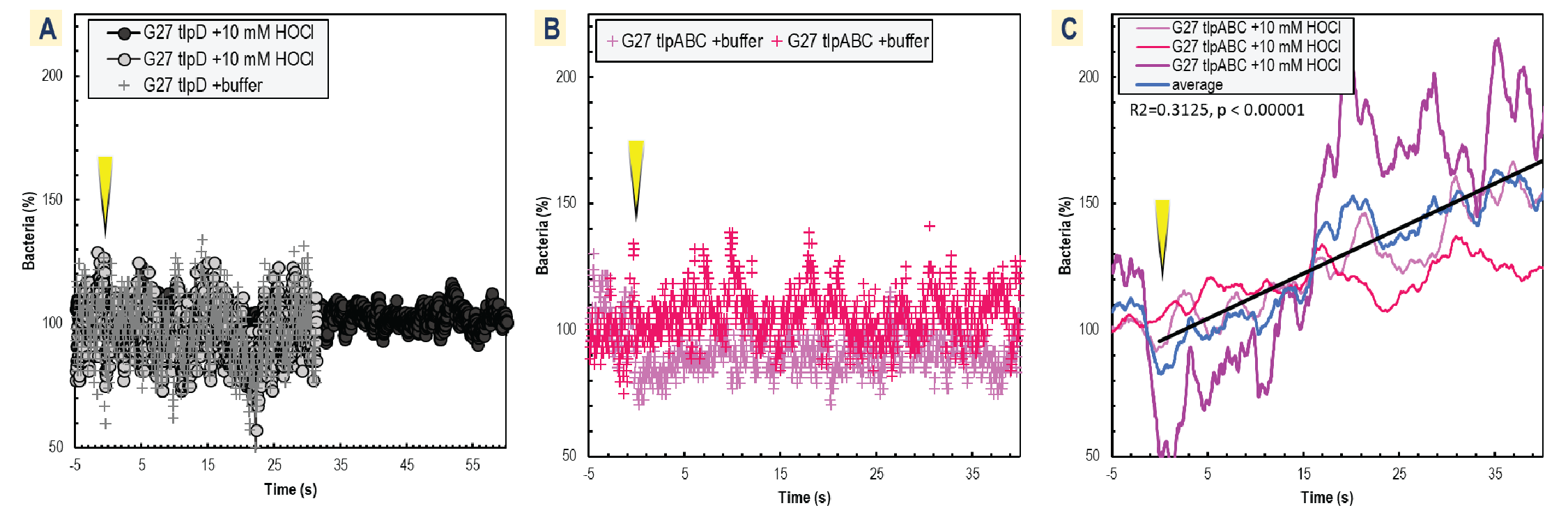

Supplement: S10 Fig — Shown are data from point-source chemotaxis assays collected on different days with G27 tlpD (A) and G27 tlpABC (B–C) mutants with either 500 mM PBS (pH 6.7; buffer) or 10 mM HOCl and 500 mM PBS (pH 6.7) in the micropipette. For clarity, data shown in (C) are smoothed by averaging normalized bacteria counts over 1 second intervals (30 frames). A linear model (black line) is fit to the post–HOCl-treated tlpABC counts over all frames, showing a positive correlation of approximately 0.3 over 40 seconds for 3 independent replicates (n = 3,600, p < 0.00001). (TIF) [file pbio.3000395.s010.tif]
