# Supplementary material for: Helicobacter pylori senses bleach (HOCl) as a chemoattractant using a cytosolic chemoreceptor
Source: PLoS Biol. 2019 Aug 29;17(8):e3000395. doi: 10.1371/journal.pbio.3000395 (PMC6715182; doi:10.1371/journal.pbio.3000395)
Supplement: S1 Table — (DOCX) [file pbio.3000395.s027.docx]

**S1 Table.** Kinetics and Dissociation Constants of Chemotaxis Complex Components.

| **Autophosphorylation^1^** | **Kinetics** |
| --- | --- |
| CheA | V_max ATP_ = 0.033 µM min^-1^, K_M ATP_ = 136 µM |
| CheA, CheW | V_max ATP_ = 0.075 µM min^-1^ |
| CheW, CheW, TlpD | V_max ATP_ = 0.43 µM min^-1^ |
| **Complex Formation^2,3^** | **Dissociation Constants** |
| TlpD monomer ↔ dimer  TlpD(-His) monomer ↔ dimer | K_D_ = 188 nM SEM=+121 nM  K_D_ = 65.7 nM SEM=+2.07 nM |
| CheA+CheW ↔ CheA-CheW | K_D_ = 14.6 µM ^4^ |
| CheA-CheW+TlpD ↔ CheA-CheW-TlpD | K_D_= 15.2 µM ^4^ |

1. Rates are for CheA autophosphorylation from data presented in Fig. 2 of either CheA alone, saturating concentrations of CheW, or maximally-activated by CheW and saturating concentrations of TlpD.
2. Parameters determined for complex formation are shown from data presented in Fig. 2. For titration of TlpD the concentration of CheA-CheW complex was estimated by titration of CheW against CheA (see Materials & Methods).
3. All proteins contain an N-terminal His-tag, except where noted by “TlpD(-His),” in which the tag was cleaved with TEV protease (see Material and Methods).
4. Due to limitations in protein solubility the maximally-saturated ends of these curves are poorly defined, and so the values reported may represent low-end estimates.
