# Supplementary material for: Helicobacter pylori senses bleach (HOCl) as a chemoattractant using a cytosolic chemoreceptor
Source: PLoS Biol. 2019 Aug 29;17(8):e3000395. doi: 10.1371/journal.pbio.3000395 (PMC6715182; doi:10.1371/journal.pbio.3000395)
Supplement: S2 Table — (DOCX) [file pbio.3000395.s028.docx]

**S2 Table.** *H. pylori* Swimming Reversal Rates in the Presence of Chemoeffectors^1^.

|  | **Mean Reversals/s** | **n** | **5^th^/95^th^ Percentile^2^** |
| --- | --- | --- | --- |
| **WT**  buffer  30 mM HCl  1 mM urea  250 µM HOCl  500 µM HOCl  1000 µM HOCl | 1.77  2.11  1.21  1.60  1.54  1.21 | 669  1314  1263  1134  1070  765 | 0.00/4.48  0.00/5.65  0.00/4.55  0.00/4.54  0.00/4.11  0.00/4.14 |
| ***cheA***  buffer  250 µM HOCl  500 µM HOCl  1000 µM HOCl | 0.32  0.54  0.41  0.36 | 810  1214  661  1120 | 0.00/1.50  0.00/2.50  0.00/2.03  0.00/1.89 |
| ***tlpD***  buffer  250 µM HOCl  500 µM HOCl  1000 µM HOCl | 0.53  0.38  0.54  0.62 | 1285  948  764  839 | 0.00/2.73  0.00/1.65  0.00/3.82  0.00/3.31 |
| ***tlpABC***  buffer  250 µM HOCl  500 µM HOCl  1000 µM HOCl | 1.47  1.12  0.99  0.63 | 731  662  212  336 | 0.00/5.40  0.00/6.42  0.00/6.40  0.00/3.03 |

1. Values are for data presented in Fig. 5D and Fig. S7B using *H. pylori* G27 wildtype and mutants.
2. Data are not normally distributed, and p-values reported were calculated using the nonparametric Kolmogorov-Smirnov two sample test, so here we report the 5^th^ and 95^th^ percentile reversal rates (s^-1^) as a nonparametric measure of the data distribution.
