# Supplementary material for: Helicobacter pylori senses bleach (HOCl) as a chemoattractant using a cytosolic chemoreceptor
Source: PLoS Biol. 2019 Aug 29;17(8):e3000395. doi: 10.1371/journal.pbio.3000395 (PMC6715182; doi:10.1371/journal.pbio.3000395)
Supplement: S3 Table — (DOCX) [file pbio.3000395.s029.docx]

**S3 Table.** Key Resources.

| REAGENT or RESOURCE | SOURCE | IDENTIFIER |
| --- | --- | --- |
| **Antibodies** | | |
| Anti-cysteine sulfenate | Kerafast | EST022 |
| Anti-TlpD | K. Ottemann (UCSC) | N/A |
| Anti-rabbit-HRP | Novus Biologicals | HAF008 |
| **Bacterial Strains** | | |
| *H. pylori* strain G27 wildtype | K. Ottemann (UCSC) | N/A |
| *H. pylori* strain G27 *tlpD* | K. Ottemann (UCSC) | N/A |
| *H. pylori* strain G27 *tlpABC* | K. Ottemann (UCSC) | N/A |
| *H. pylori* strain G27 *cheA* | K. Ottemann (UCSC) | N/A |
| *H. pylori* strain PMSS1 | M. Amieva (Stanford) | N/A |
| *E. coli* strain UU1250 | S. Parkinson (UU) | N/A |
| **Biological Samples** |  |  |
| N/A |  |  |
| Chemicals, Peptides, and Recombinant Proteins | | |
| *H. pylori* TlpD strain SS1 | Genscript | N/A |
| *H. pylori* CheW strain SS1 | Genscript | N/A |
| *H. pylori* CheA strain G27 | Genscript | N/A |
| *H. pylori* TlpD C340A strain SS1 | Genscript | N/A |
| *S. enterica* McpA strain LT2 | Genscript | N/A |
| *E. coli* DgcZ-CZB strain K12 | T. Schirmer (UB) | N/A |
| Zinpyr-1 | Abcam | Ab146332 |
| **Critical Commercial Assays** | | |
| Pierce ECL detection kit | ThermoFisher | 32106 |
| **Deposited Data** | | |
| N/A |  |  |
| **Experimental Models: Cell Lines** | | |
| N/A |  |  |
| **Experimental Models: Organisms/Strains** | | |
| *E. coli* BL21 (DE3) Arctic Cells | Agilent | 230192 |
| **Oligonucleotides** | | |
| N/A |  |  |
| **Recombinant DNA** | | |
| Plasmid pBH | Goers Sweeney et al. 2012 | N/A |
| **Software and Algorithms** | | |
| PhyML | Guindon et al. 2010 | http://www.phylogeny.fr/ |
| i-Tasser server | Zang et al. 2008 | https://zhanglab.ccmb.med.umich.edu/I-TASSER/ |
| Excel Solver | Microsoft Excel | Microsoft Corp. |
| SedFit | Lebowitz et al. 2002 | http://www.analyticalultracentrifugation.com/download.htm |
| Pathasarathy Lab Tracking GUI | Pathasarathy et al. 2012 | https://pages.uoregon.edu/raghu/particle_tracking.html |
